# Genome-scale single-cell mechanical phenotyping reveals disease-related genes involved in mitotic rounding

**DOI:** 10.1038/s41467-017-01147-6

**Published:** 2017-11-02

**Authors:** Yusuke Toyoda, Cedric J. Cattin, Martin P. Stewart, Ina Poser, Mirko Theis, Teymuras V. Kurzchalia, Frank Buchholz, Anthony A. Hyman, Daniel J. Müller

**Affiliations:** 1 Max Planck Institute of Molecular Cell Biology and Genetics, Pfotenhauerstrasse 108, 01307 Dresden, Germany; 2 Division of Cell Biology, Life Science Institute, Kurume University, Hyakunen-Kohen 1-1, Kurume, Fukuoka 839-0864 Japan; 3 Department of Biosystems Science and Engineering (D-BSSE), Eidgenössische Technische Hochschule (ETH) Zurich, Mattenstrasse 26, 4058 Basel, Switzerland; 4 Department of Chemical Engineering, Massachusetts Institute of Technology, 500 Main Street, Cambridge, MA 02139-4307 USA; 5 The Koch Institute for Integrative Cancer Research, Massachusetts Institute of Technology, 500 Main Street, Cambridge, MA 02139-4307 USA; 6 UCC, Medical System biology, Medical Faculty Carl Gustav Carus, University of Technology Dresden, Am Tatzberg 47/49, 01307 Dresden, Germany

## Abstract

To divide, most animal cells drastically change shape and round up against extracellular confinement. Mitotic cells facilitate this process by generating intracellular pressure, which the contractile actomyosin cortex directs into shape. Here, we introduce a genome-scale microcantilever- and RNAi-based approach to phenotype the contribution of > 1000 genes to the rounding of single mitotic cells against confinement. Our screen analyzes the rounding force, pressure and volume of mitotic cells and localizes selected proteins. We identify 49 genes relevant for mitotic rounding, a large portion of which have not previously been linked to mitosis or cell mechanics. Among these, depleting the endoplasmic reticulum-localized protein FAM134A impairs mitotic progression by affecting metaphase plate alignment and pressure generation by delocalizing cortical myosin II. Furthermore, silencing the *DJ-1* gene uncovers a link between mitochondria-associated Parkinson’s disease and mitotic pressure. We conclude that mechanical phenotyping is a powerful approach to study the mechanisms governing cell shape.

## Abstract (web summary)

During cell division animal cells generate intracellular pressure and round against their environment, but the genes responsible for this are largely unknown. Here the authors use a microcantilever- and RNAi-based assay to screen > 1000 genes and identify 49 genes involved in mitotic cell rounding; many are novel to this process.

## Introduction

Cell rounding is a hallmark of animal mitosis both in artificial cultures in vitro and naturally forming tissue in vivo^1, 2^. Animal cells that cannot round against extracellular confinements are inhibited in their progression through mitosis and prone to mitotic spindle defects^3–5^. In addition to facilitating the geometrical requirements of mitosis, mitotic cell rounding has been implicated in tissue morphogenesis during development^6–8^, and the maintenance of proper epithelial tissue architecture^9^.

Mitotic cells facilitate rounding by generating actomyosin-dependent surface tension and intracellular pressure^3, 5, 10–12^. Biochemically, mitotic cell rounding is regulated by the master cell cycle regulator Cdk1^13^. Cdk1 signaling oversees the reorganization of the actomyosin cytoskeleton from its interphase arrangement into a highly contractile and uniform cortex in mitosis^14^. Physically, mitotic cell rounding is driven by the generation of an intracellular pressure, which is guided into shape by the contracting actomyosin cortex^10^. The contraction increases cell surface tension mostly *via* myosin II^11^. However, owing to the Law of Laplace, actomyosin-dependent cell surface tension is transduced into intracellular pressure^15, 16^. Mitotic cells thus can employ the actomyosin cortex to balance and modulate intracellular pressure^11, 16^. This mechanism allows mitotic cells to push against neighboring impediments, such as surrounding cells or extracellular matrix, and round up against confinement^3, 10–12, 17^. Consequently, the mitotic intracellular pressure may be up to tenfold higher than that of interphase^10, 11, 16^. The actomyosin cortex and intracellular pressure together can thus be considered a macromolecular engine that transduces biochemical signals into physical action, thereby generating the mechanical forces required for cell rounding against confinement.

Although the core cytoskeletal processes associated with mitotic cell rounding are well defined, a systems level perspective of pathways supporting the mechanics of mitotic rounding is lacking. One of the problems with analyzing mechanical phenotypes is that current assays screen cellular phenotypes from a morphological rather than from a mechanical perspective. Recently introduced atomic force microscopy (AFM)-based microcantilever assays, which allow to read out the force, pressure and cortex tension generated by a rounding mitotic cell, are of low throughput, because to mechanically characterize a cell throughout mitosis requires about one hour^10, 18^. Further identification of genes required for cell rounding requires methods that greatly increase throughput of mechanical phenotyping, without losing the precision of observation. Here we scale up a recently invented microcantilever-based assay^10, 18^, by measuring the rounding force and intracellular pressure of mitotic cells at single time points, allowing the precise analysis of up to 30 cells per hour. We demonstrate the efficacy of this method by performing a genome-scale RNAi screen of > 1000 genes. After conducting the screen, we confirm 49 hits among the genes tested from which we further characterize two unanticipated hits, including a poorly characterized gene encoding for the endoplasmic reticulum (ER)-localized protein FAM134A, and a gene associated with Parkinson’s disease, *DJ-1*.

## Results

### Genome-scale mechanical phenotyping of mitotic rounding

**Fig. 1:**
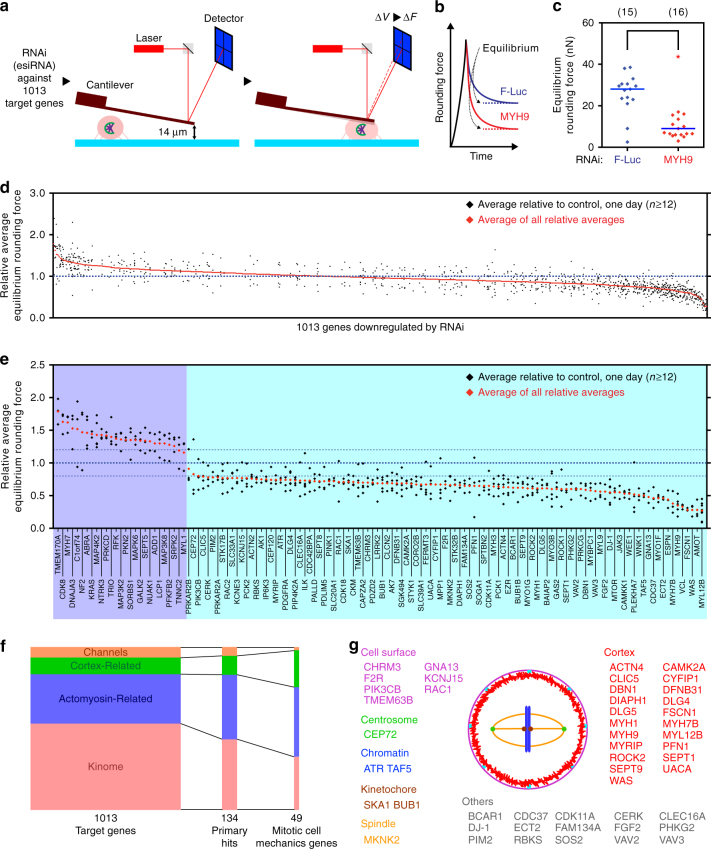
RNAi-based screen to identify genes affecting mechanical properties of mitotic cells. **a** Procedure for screening mechanical properties of mitotic cells. Cells were RNAi-transfected 48–72 h before experiments and chemically arrested in mitosis. A tipless microcantilever was used to confine ~20 µm high mitotic cells at 14 µm and to measure the response force (“rounding force”, ∆*F* in the schematic). ∆*V*, photodiode voltage measuring the force-induced cantilever deflection via a laser (red). **b** Typical time-dependent rounding force of confined mitotic cells. Traces for control (firefly luciferase, F-Luc, blue) and myosin II (MYH9, red) RNAi. Equilibrium rounding forces are achieved within 10–30 s (dashed lines). **c** Plot of equilibrium rounding forces of mitotic cells treated with F-Luc and MYH9 RNAi. Each diamond indicates rounding force of a mitotic cell. ‘(*n*)’, number of cells measured. Blue bars, mean. Statistical significance was determined by Student’s *t*-test (*p ≤ 0.05). **d** Overview of the primary screen results. The relative average equilibrium rounding force of RNAi-treated cells vs. F-Luc control RNAi-treated cells is plotted. At least 12 cells were analyzed per condition. The genes (*x* axis) are ordered by the average relative force (red). Blue dotted line denotes average relative equilibrium rounding force for control cells. See Supplementary Fig. 2 for screen workflow and Supplementary Data for full results. **e** Primary hit genes (134/1013) with relative equilibrium rounding forces. At least 12 cells were analyzed per condition. Blue dotted lines denote average (thick line), 80 and 120% of average (thin lines) for control cells. Dark blue and light blue backgrounds denote hits repeatedly leading to statistically significant increased and decreased relative equilibrium rounding forces, respectively. **f** Portion of genes belonging to the indicated gene class in the initial set of target genes, primary hits and mitotic cell mechanics genes. Relative size of rectangles represents the number of genes. **g** Subcellular localization of the 49 mitotic cell mechanics genes according to the literature^24, 25^. Red barbed line, cell cortex; magenta, cell surface; pale blue, plasma membrane-cortex linker; green, centrosome; blue, chromatin; brown, kinetochore; orange, mitotic spindle; ‘others’ include cytoplasm, mitochondria, endoplasmic reticulum, and uncharacterized

Previously we established assays to characterize the mechanical properties of mitotic cells and the forces involved in cell rounding^10, 18, 19^. These assays, which rely on a microcantilever to confine a mitotic cell and to characterize in detail how a selected protein affects the mechanical rounding of the cell, allow the characterization of about six cells progressing through mitosis per day (Supplementary Fig. 1a–c)^10, 18^. To adapt our approach for genome-scale screening, we searched for ways to increase the experimental throughput of our microcantilever-based assay. Therefore, we took the following steps: First, we increased the mitotic index, by employing *S*-trityl-l-cysteine (STC), an inhibitor of a mitotic kinesin Eg5/KIF11^20^. Mitotic arrest by STC or inhibitors of polo-like kinase 1 (PLK1) or aurora kinase A did not significantly alter mitotic cell mechanics, as defined by measurements of rounding force or pressure (Supplementary Fig. 1d–j). Second, we developed a procedure for measuring the equilibrium rounding force in arrested mitotic cells. An AFM-microcantilever was moved to confine a mitotic cell at a given height of 14 µm, kept in place until the force equilibrated, and then moved onto the next cell (Fig. 1a, b, Supplementary Fig. 2a–e). The equilibrium force together with the midplane image of the confined mitotic cell was used to determine cell geometry and thereby calculate intracellular pressure^10, 16^.

Next, we sought to screen the mitotic-specific mechanical phenotypes of greater than 1000 genes. A library of endoribonuclease-prepared small interfering (esi)RNAs^21^ targeting 1013 genes that are potentially involved in the implementation or regulation of mitotic cell rounding was compiled. We have chosen esiRNAs because they show substantially less off-target effects compared to chemically synthesized siRNAs^21, 22^. The library consisted of esiRNAs targeting cortex- and actomyosin-related genes, membrane-associated transporters and kinases (Supplementary Data 1, see Methods for the criteria of gene selection). We routinely confirmed efficacy of our transfection protocol by establishing a daily positive control based on myosin II depletion. Knockdown of the myosin II subunits myosin heavy chain 9 (MYH9) or myosin regulatory light chain 2 (MRLC2/MYL12B) decreased rounding force by up to 90% (Fig. 1c and Supplementary Fig. 2f–i), consistent with previous reports^11, 23^.

From the 1013 esiRNAs tested, we found 134 genes (‘primary hits’) whose silencing significantly decreased or increased mitotic rounding force compared to control cells by > ± 20%, at a success rate higher than 50% of at least two (up to eight) rounds of measurements (see Methods; Fig. 1d, e and Supplementary Data 2). The cut-off of > ± 20% change in mitotic rounding force allowed to focus on genes yielding strong phentopyes. Next, a secondary set of non-overlapping esiRNAs was synthesized, targeting 124 of the 134 primary hit genes. We obtained a list of 49 genes (‘mitotic cell mechanics genes’), which reproducibly led to the same mechanical phenotype, exhibiting a > 20% reduction of rounding force when silenced (Fig. 1f and Supplementary Data 3 and 4), while none of the primary hits increasing the rounding force was confirmed in the secondary screen. From these 49 mitotic cell mechanics genes we further determined cell volume and intracellular pressure by measuring the cross-sectional area at the cellular midplane and applying a verified model assuming semi-circular side profiles of confined rounded cells (Supplementary Fig. 2j, k and Supplementary Data 4 and Supplementary Table 1)^16^. From the 49 mitotic cell mechanics genes, 34 affected cell pressure and three cell volume, whereof one gene was required for both regular pressure and volume of mitotic cells (Supplementary Fig. 3). The remaining 13 genes decreased rounding force without strongly changing cell volume or pressure.

**Fig. 2:**
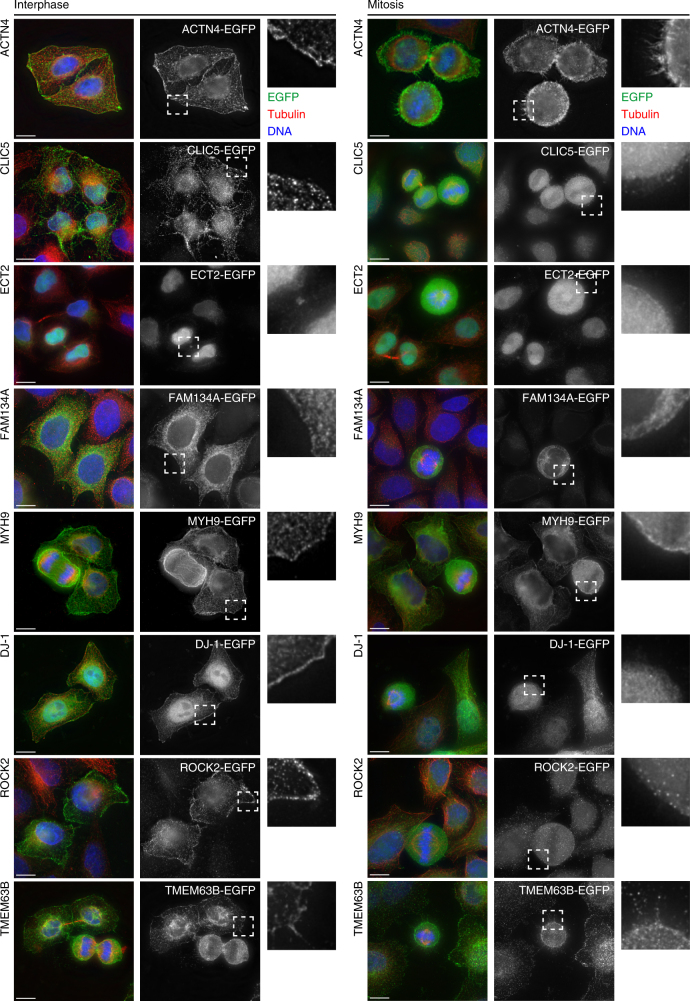
Analysis of subcellular localization of selected mitotic cell mechanics proteins. Immunofluorescence images of fixed HeLa interphase (left) and mitotic (right) cells expressing selected EGFP-tagged proteins, which were identified to result in decreased relative equilibrium rounding force when downregulated by RNAi. These proteins include ACTN4, CLIC5, ECT2, FAM134A, MYH9, DJ-1, ROCK2 and TMEM63B. Dashed boxes show the location of the EGFP-tagged protein at the cell cortex at higher magnification. Blue, DNA (DAPI); Green/grey, EGFP (goat anti-GFP antibody/Alexa-488); Red, tubulin (mouse anti-alpha-tubulin/Alexa-594). Other relevant proteins had been tagged with EGFP using BAC TransgeneOmics^26^ and are analyzed in Supplementary Fig. 4. Scale bars, 10 µm

According to current literature^24, 25^, the proteins encoded by the 49 mitotic cell mechanics genes exhibit a wide range of subcellular localizations (Fig. 1g). To localize the proteins in interphase and mitosis, HeLa cells stably expressing the tagged transgenes were made by the BAC TransgeneOmics technology^26^ and assessed by immunofluorescence. Most tagged proteins localized at the cell cortex either in interphase or mitosis (Fig. 2 and Supplementary Fig. 4), thus confirming the well-known importance of the cell cortex in mitotic cell rounding. However, ECT2 and DJ-1 were observed at the nucleus and FAM134A coincided with an ER-like meshwork (Fig. 2). This variation of protein localization suggests that mitotic cell mechanics is underpinned by a diverse set of biological processes.

We next placed the proteins of the 49 mitotic cell mechanics genes into known pathways. Among them were myosin subunits, actin-bundling proteins and components of the RhoA small GTPase cascade^27^, such as alpha-actinin-4 (ACTN4), fascin 1 (FSCN1), ECT2 and Rho kinase (ROCK), consistent with previous reports^17, 28–31^. From the screened membrane-associated proteins, an intracellular chloride channel CLIC5 and the inwardly rectifying potassium channel KCNJ15 were identified. Further, testing the full human kinome allowed us to isolate various protein-, lipid- and carbohydrate kinases, which were mostly hitherto not connected to cell mechanics. A spindle checkpoint kinase BUB1^32^ was unexpectedly isolated, suggesting a potential link between the kinetochore and cell rounding. The screen did not extensively enrich for a set of ‘cell division genes’ defined by previous RNAi screens^33, 34^, suggesting that, under our in vitro screening conditions, a specific set of genes is required for mitotic cell rounding distinct from those required for cell division (Supplementary Fig. 3b). Lastly, one of the 49 mitotic cell mechanics genes, *DJ-1*, showed association with Parkinson’s disease^35^, suggesting the potential usefulness of our assays to discover novel functions of genes associated with neurodegeneration.

In the next sections, we survey some of the different classes of genes that are required for cell rounding, focusing on genes required to target myosin II and genes that are required for mitotic-specific cell pressure generation. Finally, we focus on the role of one gene of hitherto unknown function, *FAM134A*, and one implicated in Parkinson’s disease, *DJ-1*.

### Diverse genes target myosin II to the mitotic cell cortex

**Fig. 3:**
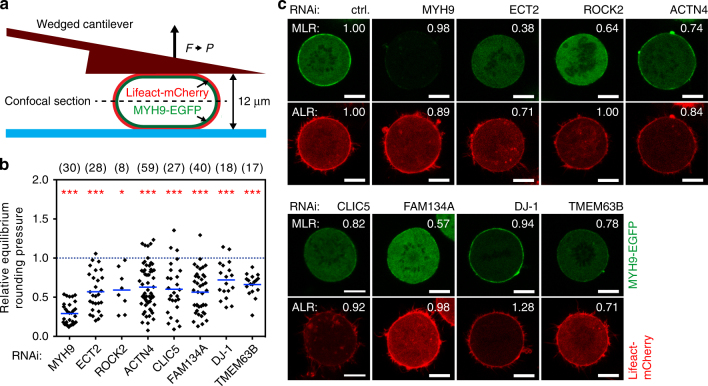
Depletion of mitotic cell mechanics genes is linked to impaired cortical targeting of myosin II. **a** Depiction of the assay to measure rounding pressure of mitotic cells. STC-arrested mitotic cells were confined to a set height of 12 µm by a microcantilever equipped with a terminal wedge. Rounding pressure *P* was calculated from force *F* and contact area derived from images at the cross-sectional midplane (Methods). Cortex to cytoplasm ratio of fluorescence markers for myosin II (*MLR* myosin localization ratio) and actin (*ALR* actin localization ratio) were calculated as described in Methods^11^. **b** Relative equilibrium rounding pressure of STC-arrested confined mitotic cells depleted of the indicated proteins by RNAi. Blue dashed line, average control rounding pressure, normalized to one. Each black diamond represents one cell. ‘(*n*)’ denotes number of cells measured. Blue bars, mean. Statistical significance was determined by Mann–Whitney *U*-test (*p ≤ 0.05; ***p < 0.001). **c** Representative fluorescent confocal images of STC-arrested confined mitotic cells expressing markers for myosin II (MYH9-EGFP) and F-actin (Lifeact-mCherry), depleted of selected proteins as indicated. MLR and ALR, relative to control cells, are shown at the top right of each image. Scale bars, 10 µm. See Supplementary Fig. 5 for full results of rounding pressure and MLR/ALR for all genes tested

We have recently shown that recruitment of myosin II to the mitotic cortex scales directly with the pressure generated by the rounding cell^11^. To analyze whether the mechanical phenotypes uncovered in our RNAi screen are linked to cytoskeletal perturbations, we selected eight genes (*MYH9*, *ECT2*, *ROCK2*, *ACTN4*, *CLIC5*, *FAM134A*, *DJ-1*, *TMEM63B*) and examined the localization of their protein products in HeLa cells stably expressing markers for myosin II (MYH9-EGFP) and F-actin (Lifeact-mCherry or Actin-EGFP). This allowed the simultaneous measurement of intracellular pressure and imaging of myosin II and F-actin (Fig. 3a). By image analysis, actin/myosin fluorescence intensity ratios of the cortex to the cytoplasm (ALR/MLR for actin/myosin localization ratio, respectively) were calculated for each mitotic cell^11^. RNAi of six of the eight genes (75%) impaired the cortical localization of myosin II (Fig. 3b, c and Supplementary Fig. 5): *ECT2*, *ROCK2*, *ACTN4*, *CLIC5*, *FAM134A* and *TMEM63B*. These genes, except *ROCK2* and *FAM134A*, also affected the cortical localization of F-actin.

Roles for ECT2 and ACTN4 in cortical targeting of myosin II have been reported^17, 36^. Moreover, the involvement of Rho kinases (ROCK1 or ROCK2) in mitotic cell rounding was previously demonstrated^11, 31^. Our results thus confirm that the mechanical phenotypes of these genes are related to impaired actomyosin cortex integrity, either by weakening the composition of the actin meshwork or by directly interfering with myosin II targeting to the mitotic cortex. The intracellular chloride channel CLIC5 has been shown to associate with, and to be regulated by, the actin cytoskeleton^37, 38^. It is also linked to cytoskeletal processes involved in podocyte integrity and cell stereocilia stability^39, 40^. As depletion of CLIC5 led to decreased cortical targeting of actin and myosin II (Fig. 3b, c and Supplementary Fig. 5e), our observation suggests a role for CLIC5 in organizing the mitotic cell cortex.

We were further intrigued by the finding that the depletion of transmembrane protein 63B (TMEM63B), a putative plasma membrane cation channel required for cell division^24, 33^, led to decreased cortical targeting of F-actin and myosin II and, consequently, impaired cell pressure (Fig. 3b, c and Supplementary Fig. 5h). This points to a link between regulation of ion transport and the integrity of the actomyosin cytoskeleton. Depletion of MYH9 did not alter the ratio of cortical to cytosolic myosin II but substantially decreased the overall level of MYH9-EGFP fluorescence, thus indicating a generally decreased amount of myosin II. Finally, depletion of FAM134A affected cortical myosin targeting, while depletion of DJ-1 did not.

### FAM134A supports mitotic progression and cell pressure

We next screened for genes that had a mitosis-specific role in building up of intracellular pressure. For this purpose, we picked 12 genes from the screen whose downregulation by RNAi reproducibly decreased mitotic cell pressure, and measured the interphase pressure by trypsinizing interphase cells and confining them between a microcantilever equipped with a polymer wedge and the glass substrate (Supplementary Fig. 6a–d). This ‘parallel plate’ configuration was necessary to stably isolate trypsinized cells for mechanical phenotyping^5^. The assay identified eight genes (67%) that were required for cell pressure both in mitotic and interphase cells (Supplementary Fig. 6e–p). However, cells depleted of ECT2, FAM134A, FSCN1, and SLC33A1 solely showed mitosis-specific mechanical phenotypes.

**Fig. 4:**
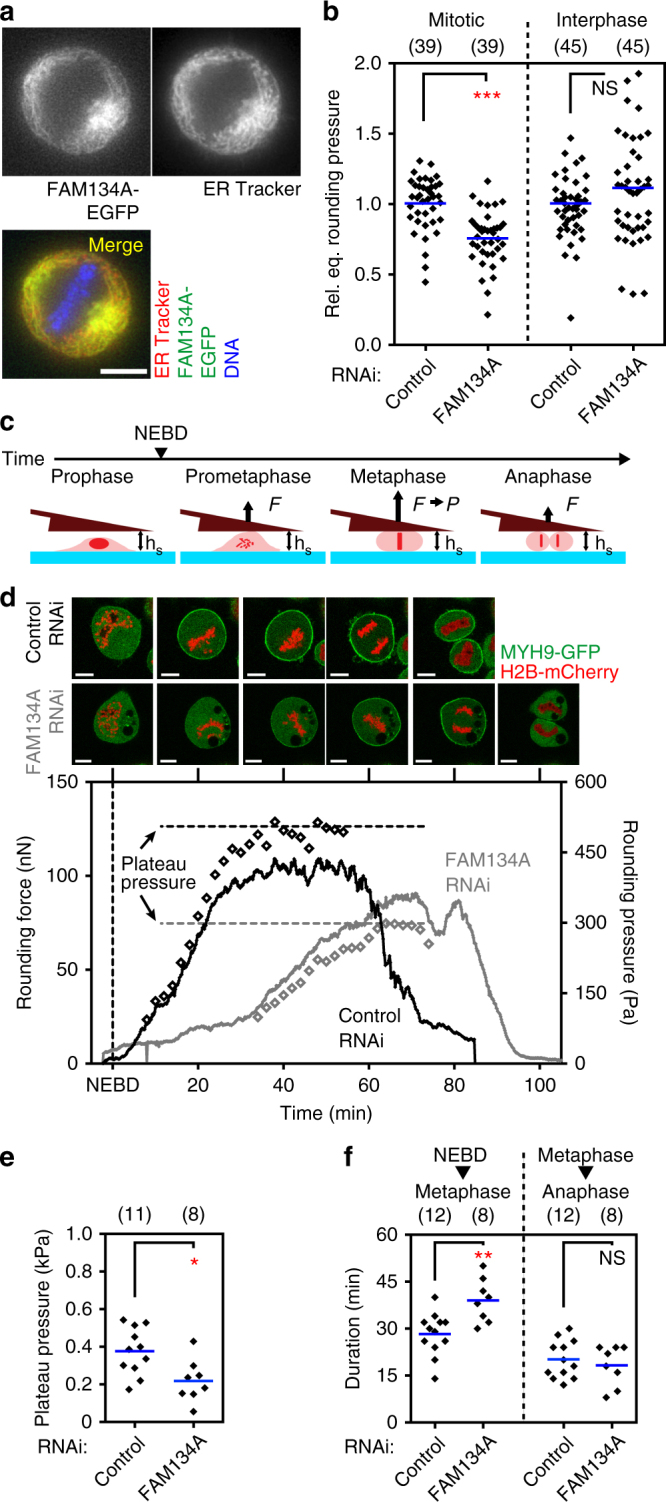
The endoplasmic reticulum (ER) protein FAM134A is required for rounding pressure during mitosis and timely prometaphase progression. **a** Co-localization of human FAM134A-EGFP and an ER marker in mitosis. Blue, DNA; Green, FAM134A-EGFP; Red, ER Tracker, an ER marker. Yellow regions represent co-localization between FAM134A and ER. Scale bar, 10 µm. **b** Relative equilibrium (Rel. eq.) rounding pressure of mitotic and interphase cells upon depletion of FAM134A by RNAi. Values relative to average rounding pressure of control are shown. See Supplementary Fig. 6 for a description of the assay used and for full results for all genes tested. **c** Depiction of the trans-mitotic height confinement assay used to determine the intracellular pressure of a cell rounding against a wedged cantilever (brown) held at a height *h*_s_ of 10 µm. *P*, rounding pressure; *F*, rounding force. Blue, glass surface. **d** Representative confocal microscopy images (top) and force traces (bottom) of H2B-mCherry and MYH9-EGFP expressing RNAi treated HeLa cells submitted to the assay depicted in **c**. Lines represent force traces, open diamonds represent rounding pressure values. NEBD, nuclear envelope breakdown. Scale bars, 10 µm. **e** Plateau pressure of metaphase cells as depicted in **d**. **f** Duration from NEBD to metaphase plate formation (left) and from metaphase plate formation to anaphase onset (right) for cells submitted to the assay depicted in **c**. Each black diamond represents one cell. ‘(*n*)’ denotes number of cells measured. Blue bars, mean. Statistical significance was determined by Mann–Whitney *U*-test (NS, *p* > 0.05; *p ≤ 0.05; **p < 0.01; ***p < 0.001)

Because FAM134A was required for intracellular pressure only in mitosis and for targeting myosin II to the cortex (Figs. 3c and 4b), we further investigated its role in cell rounding. First, we wanted to confirm the mechanical phenotype observed in our screen, which used two independent esiRNAs (Supplementary Table 1). We thus repeated mechanical phenotyping using four different siRNA duplexes targeting FAM134A. H2B-EGFP and mCherry-CAAX expressing HeLa cells treated with three of the four oligonucleotides showed a decreased mitotic rounding force (Supplementary Fig. 7a). Moreover, FAM134A-EGFP expressing HeLa cells treated with the two independent esiRNAs and three of the four siRNA oligonucleotides showed a decreased mitotic rounding pressure (Supplementary Fig. 7b). Second, we were curious about the cell-cycle-specific localization of FAM134A, as a mitotic function has not previously been ascribed to it. Using a cell line expressing FAM134A-EGFP, we observed that it was localized to a tubule-like meshwork roughly outside the spindle apparatus in mitosis. FAM134A largely co-localized with an ER marker, but not with mitochondria (Fig. 4a and Supplementary Fig. 7c–f). Third, consistent with its tubular localization, endogenous FAM134A was detected in the membrane fraction in a cell fractionation experiment (Supplementary Fig. 7g). Fourth, we confirmed defective targeting of myosin II to the mitotic cortex as well as the decreased pressure in FAM134A-depleted cells progressing through mitosis (Fig. 4c–e). Fifth, we found a delay in metaphase plate alignment in these FAM134A-depleted cells (Fig. 4f and Supplementary Movies 1 and 2). Together these results indicate that FAM134A is an ER-associated protein involved in mitotic spindle fidelity and recruitment of myosin II to the mitotic, but not the interphase, actin cortex.

### Enzymatic function of DJ-1 underpins mitotic cell pressure

**Fig. 5:**
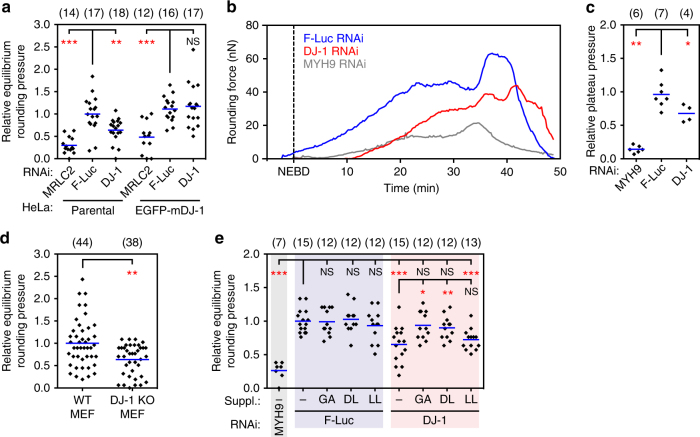
Glycolate and d-lactate rescue the impaired intracellular pressure of mitotic DJ-1-depleted cells. **a** Rescue of the decreased mitotic rounding pressure phenotype of HeLa cells by a mouse DJ-1 transgene. HeLa cells were treated with RNAi targeting DJ-1, myosin regulatory light chain (MRLC2) or no human gene (firefly luciferase control, F-Luc). Rounding pressure relative to average F-Luc control pressure of cells without transgene (parental). **b** Representative rounding force traces for HeLa cells RNAi treated as indicated (for a depiction of the assay see Fig. 4c). NEBD, nuclear envelope breakdown. **c** Plateau pressure during metaphase. Values relative to average F-Luc control values. **d** Rounding pressure of STC-arrested wild type (WT) and DJ-1 knockout (KO) mouse embryonic fibroblasts (MEFs). Rounding pressure relative to average WT MEF pressure. **e** Enantiomer-specific rescue of the pressure defect caused by DJ-1 knockdown. *GA* glycolate; *DL* d-lactate; *LL* l-lactate. Cells were supplemented with 1 mM of indicated chemicals for 1 h prior to measurements. Values relative to average F-Luc control values. Each black diamond represents one cell. ‘(*n*)’ denotes number of cells measured. Blue bars, mean. Statistical significance was determined by Mann–Whitney *U*-test (NS, *p* > 0.05; *p ≤ 0.05; **p < 0.01; ***p < 0.001)

One striking result from the present screen was that the Parkinson’s disease-associated gene *DJ-1/PARK7* ^35^ decreased mitotic cell pressure upon silencing by RNAi. Since this phenotype manifested even without affecting the cortical targeting of myosin II (Supplementary Fig. 5g), we further investigated the influence of DJ-1 on mitotic cell rounding. First, to confirm that the low-pressure phenotype observed upon RNAi-treatment is indeed linked to depletion of DJ-1, we expressed the mouse DJ-1 transgene (EGFP-mDJ-1) in HeLa cells. We found that in HeLa cells expressing EGFP-mDJ-1, the low-pressure phenotype found in parental cells upon depletion of DJ-1 by RNAi was rescued (Fig. 5a). Second, DJ-1-depleted HeLa cells showed not only decreased rounding force and pressure in the STC-arrested state but throughout mitosis (Fig. 5b, c). Third, we tested the effect of knocking out the *DJ-1* gene in mouse embryonic fibroblasts (MEFs)^41^, which led to considerably lower pressure during mitosis compared to wild type MEFs (Fig. 5d and Supplementary Fig. 8). These additional experimental findings further strengthened the link between DJ-1 and mitotic cell mechanics.

*DJ-1* is highly conserved from bacteria to higher eukaryotes^42^, and it was originally reported as an oncogene, expressed in most tissues^43^. Recently, DJ-1 was reported to represent a novel class of glyoxalases (GLO III), producing alpha-hydroxyacids (e.g. glycolic acid and D-lactic acid) from alpha-oxoaldehydes (e.g., glyoxal and methylglyoxal)^44^. We and others have previously shown that glycolate (GA) and D-lactate (DL) restore the decreased mitochondrial membrane potential measured upon silencing *DJ-1* or another Parkinson’s disease-related gene, *PTEN-induced putative kinase 1* (*PINK1*)^45–47^. We therefore wanted to investigate whether GA and DL could rescue the phenotype of decreased mitotic cell pressure. We first determined the most effective concentration of GA and DL by exogenous addition to *DJ-1*-downregulated mitotic cells (Fig. 5e and Supplementary Figs. 9 and 10). Addition of 1 or 10 mM GA or DL restored the cell pressure defect caused by *DJ-1* downregulation in HeLa cells and in *DJ-*1-knockout MEFs after one hour of incubation, while the effect of 0.1 mM GA in HeLa was not significant. l-lactate (LL), however, did not restore the mechanical defect, indicating an enantiomer-specific rescue (Fig. 5e). These observations infer that the absence of the products of DJ-1 lowers cell pressure and thus causes the mechanical phenotype without affecting mitotic myosin II targeting.

## Discussion

The use of large-scale loss of function screens has been a powerful approach to hunt for genes involved in phenotypes detected by high-throughput optical microscopy^48, 49^. While much has been learned from phenotypes that are lethal, in many cases, phenotypes are subtle, and do not lead to obvious defects in survival. This has two consequences. There are many genes in the genome the function of which is not annotated; and there are many genes that are implicated in disease, but which have little obvious defects in model systems. Therefore, complementary parameters to probe cellular function at genome-scale throughput are needed. Here, using a simple microcantilever-based assay to detect force and pressure generated by rounded mitotic cells, we have screened more than 1000 genes for those that affect the cell rounding force and pressure generated during mitosis. Out of these genes we found 49 that lead to mechanical phenotypes when silenced. Whereas all 49 genes decreased the rounding force generated by mitotic cells, 36 exhibited changes in volume and/or intracellular pressure.

Although mitotic cell rounding has been implicated in maintenance of tissue architecture in vivo, generation of mitotic cell pressure is unlikely to be important for cell survival in tissue culture in vitro^6–8^. This is presumably why many of the hits we have identified have not scored positive in the previous large-scale RNAi screens examining mitotic function. More generally, one common problem in analyzing the function of genes that, for instance, have been implicated in neurodegenerative disease is that their dysfunction often does not result in an obvious consequence in mice or in tissue culture^50^. This is presumably because genes whose mutations appear later in life have only minor effects on cell physiology. It therefore seems likely that assays that look at the more subtle or non-lethal defects in cell physiology will be necessary for uncovering the function of these genes. This suggests that a strategy based around large-scale mechanical phenotyping could reveal a wealth of as yet unstudied cell biology and its relationship to disease.

Mitotic cell rounding has been observed in a diverse set of organisms and occurs in almost all eukaryotic cells, which implies an important role for this process in cell division^2, 4^. For example, when human epithelial cells are confined to heights below seven micrometers and hence hindered from rounding in mitosis, mitotic progression is impaired^3^. Cell rounding in mitosis is driven by an increase in contractility of the actomyosin cortex that is balanced by intracellular osmotic pressure^10, 51^. Previous studies have shown that formin-mediated F-actin nucleation and Rho-based myosin II contractility are central to mitotic cell rounding^11, 17, 52^. As expected, many of our intracellular pressure phenotypes arise from defective myosin II targeting to the mitotic cortex. Among the screened channel proteins that were previously shown to be membrane-associated, an intracellular chloride channel, CLIC5, and the inwardly rectifying potassium channel KCNJ15 as well as a putative cation channel, TMEM63B, were identified, suggesting that our cell mechanical screen is also a powerful way to identify novel mechanisms the cell uses to maintain intracellular pressure. Interestingly, these hits indicate that membrane proteins are key players in the mechanics of mitosis^51, 53, 54^, a role which has until recently been assigned mainly to proteins of the cytoskeleton. Follow-up studies may elucidate the exact roles of the membrane proteins found in our screen in regards to their regulation of mitotic rounding pressure.

A prominent discovery from this screen is that the products of *DJ-1*, a gene implicated in the onset of Parkinson’s disease, are required for the rounding of cells during mitosis. Generation of rounding pressure at mitosis requires an increase in osmotic pressure^10^, and it therefore seems possible that *DJ-1* mutant cells cannot generate the intracellular pressure required to round up for mitosis because they cannot maintain their osmotic pressure. We were unable to see an obvious defect on organization of F-actin or myosin II in *DJ-1*-downregulated mitotic cells in contrast to other genes that we found in our screen. One explanation is that oxidative damage incurred in the absence of DJ-1 expression might affect the function, but not localization, of actomyosin proteins or their upstream activators^35, 55^. On the other hand, DJ-1 was reported to represent a novel class of glyoxalases, producing alpha-hydroxyacids from alpha-oxoaldehydes^44^. Our follow-up experiments infer that the absence of such enzymatic products of DJ-1 lowers the mitotic cell pressure, and that the addition of these products can restore mitotic pressure, thereby removing the mechanical defect. The enzymatic products of DJ-1 GA and DL can be produced in the cytoplasm by a glyoxalases shunt of glycolysis^56^ and/or in mitochondria by D-lactate-specific lactate dehydrogenase^57^. GA and DL can support the mitotic rounding pressure by retaining the mitochondrial membrane potential^47^. Future work will be required to elucidate the cell biological basis linking mitochondrial potential and the onset of cell pressure. Left unknown is for example whether and how the effect on cell pressure by deletion of DJ-1 is linked to the onset of PD, which results from the death of dopaminergic neurons. It is thus interesting to speculate whether defects in intracellular pressure of mitotic cells could also affect viability of dopaminergic neurons. Dopaminergic neurons are particularly active and energy-consuming, due to their continuous pacemaking and unmyelinated axons, and it is possible that they are therefore uniquely sensitive to changes in cell pressure. Moreover, that a prominent disease-related gene involved in the synthesis of these products causes a mechanical phenotype may point to a unique role of similar disease-related genes in basic processes involving mitosis.

In conclusion, in this paper, we bring together two powerful methods in cell biology: genome-scale phenotypic screening, and mechanical measurements of mitotic cell pressure. Our assay allows measuring about 30 cells per hour, but future changes in methodology that take advantage of recent high-thoughput techniques should allow mechanical screening of the entire genome. Such techniques may include pillar-based confinement methods^12^, transit time through microfluidic constrictions^58, 59^, acoustic impedance analysis^60^, or hydrodynamic deformability cytometry^61, 62^. Indeed, high-throughput mechanical phenotyping has been proposed for biomedical applications such as disease diagnosis in the coming decades^63^. In the context of cell cycle analysis, we anticipate that more sensitive microcantilever-based assays^64, 65^ may be soon applied to detect subtle changes in cell pressure and mechanics related to cell states other than mitosis. The fact that this screen has identified many genes involved in diseases suggests that further screening of mechanical phenotypes of various cell states will be a powerful tool to investigate the role of disease genes or pathogenic processes across cell populations. We anticipate that large-scale single-cell mechanical phenotyping may become a key approach for analyzing gene function and characterizing disease states.

## Methods

### Cell culture

HeLa Kyoto cells stably expressing histone H2B-EGFP and mCherry-CAAX (clone 2B4), MYH9-EGFP^66^, Actin-EGFP, MYH9-EGFP and H2B-mCherry, MYH9-EGFP and Lifeact-mCherry (kindly provided by Dr. Ewa Paluch), their parental cells, and HeLa Kyoto cells expressing mouse DJ-1 transgene or FAM134A-EGFP as well as mouse embryonic fibroblasts (MEFs) were cultured in DMEM (4.5 g l^−1^ glucose, Life Technologies) supplemented with 10% fetal bovine serum (FBS), 2 mM GlutaMAX, 100 U ml^−1^ penicillin, 100 µg ml^−1^ streptomycin, plus additional antibiotics for the transgene (0.5 mg ml^−1^ Geneticin for H2B-EGFP, Actin-EGFP, MYH9-EGFP, FAM134A-EGFP, mouse DJ-1 BAC transgene and further BAC-transgenic lines^26^ shown in Fig. 2 and Supplementary Fig. 4; 0.5 µg ml^−1^ puromycin for mCherry-CAAX, H2B-mCherry and Lifeact-mCherry), at 37 °C in a 5% CO_2_ environment. For measurement of mitotic cell force/pressure, CO_2_-indepenedent DMEM containing 4 mM sodium bicarbonate and 20 mM HEPES was used. To enrich for mitotic cells, 2 µM (+)-*S*-trityl-l-cysteine (STC, Sigma-Aldrich) was added to the microscopy medium two hours prior to measurements.

### Primary and secondary screen of mitotic cell mechanics

The genes to screen (Supplementary Data 1) were defined by the following criteria: (1) proteins that localized to the cell surface in metaphase and/or telophase in a previous localization-based screen^24^; (2) genes with the GeneOntology annotation “actin cytoskeleton” found in the UniProt KB^67^; (3) all kinase genes (kinome); (4) selected channel or transporter genes; (5) genes shown in the KEGG pathway “Regulation of actin cytoskeleton” (map04810)^68^. EsiRNAs for 1013 genes were prepared in total. The clone 2B4 HeLa cells were seeded onto a glass-bottom dish (FD35-100, World Precision Instruments). On the next day, cells were transfected with 60 nM esiRNA (MISSION esiRNA, Sigma) using oligofectamine reagent (Life Technologies). On the day of measurement, typically after 48 h from transfection, culture medium was replaced with the CO_2_-independent medium, then the culture dish was mounted on a manual or an automated precision stage (JPK Instruments) on a light microscope (AxioObserver Z1 or Axiovert200, Zeiss) equipped with a ×20 objective (Plan Apochromat, NA = 0.80). The micropscopy equipment was placed in a custom-made temperature-stabilized box at 37 °C (The Cube, Life Imaging Services). Alternatively, a dish heater (JPK Instruments) was used to maintain the cells at a constant temperature of 37 °C. To measure rounding force of a mitotic cell, a tipless microcantilever (NSC12/CSC37-B with a nominal spring constant of 0.3 N m^−1^, MikroMasch, Estonia) mounted to the AFM (NanoWizard I or II or CellHesion 200, JPK Instruments) was used^18^. Cantilever calibration was done before every experiment by measuring the deflection sensitivity against the rigid glass surface followed by spring constant determination using the thermal noise method^69^. In the force measurement illustrated in Fig. 1a and Supplementary Fig. 2a, the cantilever was set at 14 µm above the glass dish and moved over the mitotically synchronized cell to measure the equilibrium force. To dispense with the need to analyze optical images, we used rounding force as a proxy for intracellular pressure. This approach allowed measuring roughly one cell per minute, thereby increasing the experimental throughput almost 100-fold compared to trans-mitotic measurements (Supplementary Fig. 2)^18^. If silencing of a gene significantly increased or decreased rounding force (*p* ≤ 0.05 in a Student’s *t*-test, and more than 20% lower/higher rounding force) compared to control RNAi using firefly luciferase esiRNA (F-Luc) in a first round of experiments measuring 12 or more cells, additional rounds of measurements (up to eight) were carried out. Based on our previous report^10^, a sample size of 12 cells was determined to be sufficient to robustly detect statistically significant differences to the control RNAi cells. Cells with obviously abnormal size, for instance due to polyploidy, were not measured. For statistical analysis of the screening data, a Student’s *t*-test was used, as data from control RNAi cells showed normal distribution as tested on Prism 5 software (GraphPad Inc.). If the same phenotype at a success rate higher than 50% of at least two rounds of measurements was found, the gene was regarded as a primary hit.

For the secondary screen, secondary esiRNAs targeting a cDNA region distinct from that of the primary esiRNA were designed^70^, synthesized and transfected as in the primary screen. In the secondary screen, volume and intracellular pressure of the mitotic cells was calculated using the maximal cross sectional area of the compressed cell from the DIC or mCherry-CAAX as described below^16^. For all genes, at least two rounds of measurements were carried out. If the same phenotype as in the primary screen was found with the conditions described above (*p* ≤ 0.05 in a Student’s *t*-test in > 50% of at least two measurements rounds, with changes higher than 20% (force, pressure) or 10% (volume)), the respective gene was considered a gene causing a mechanical phenotype in mitosis (‘mitotic cell mechanics gene’).

### Quantification of cell pressure and volume

Geometrical parameters were calculated based on the assumption of a rounded cell obeying a cortical-shell, liquid-core model in which the Young-Laplace relation between surface tension and pressure difference across the cell boundary holds true. It was shown previously that the assumption of circular side profiles serves as a decent approximation for the shape of confined mitotic cells^16^. The AFM allows reading out the force (*F*) leading to cantilever deflection and the cantilever height (*h*) above the substrate. Using the measurement tools of AxioVision and ZEN software (Zeiss) allows determining cellular midplane cross sectional area (*A*_m_), corresponding radius (*r*_m_) and distance from cantilever tip to cell edge (*x*) from images of confined cells. For all experiments, parallel plate configuration was assumed. When using conventional non-wedged cantilevers, the difference between cantilever height above the substrate at the tip of the cantilever to the actual height between the glass dish and the cantilever at the center axis of the cell was corrected for (Supplementary Fig. 2j), and so was the force-dependent height change due to cantilever deflection in all cases. Assuming parallel plate configuration for non-wedged cantilevers will induce an error when calculating shape parameters and cell pressure, which was considered negligible in light of the fact that we use these data for comparative means only. For all conditions, the relation between midplane cross sectional area (*A*_m_) and corresponding radius (*r*_m_) is

1 $${A_{\rm{m}}} = 2\pi r_{\rm{m}}^2$$

and the cell height at the center axis when using non-wedged cantilevers can be calculated as

2 $$h = {h_{{\rm{set}}}} + \frac{F}{k} + {\rm{tan}}\,10^\circ \times \left( {{r_{\rm{m}}} + x} \right)$$

where *h*_set_ is the preset cantilever height and *k* the cantilever spring constant. For wedged cantilevers (Supplementary Fig. 2k), the corresponding formula for the calculation of cell height is the same as above, apart from the last term (and corresponding determination of the distance from cantilever tip to cell edge, *x*) becoming obsolete due to the true parallel plate configuration. For long-time experiments, drifts in cantilever height and force signal where determined at the end of the experiment and recorded values were corrected accordingly, assuming linear drift over time. For all results, the pressure data were calculated using

3 $${r_{\rm{c}}} = {r_{\rm{m}}} - \frac{h}{2}$$

4 $${A_{\rm{c}}} = \pi r_{\rm{c}}^2$$

and

5 $$P = \frac{F}{{{A_{\rm{c}}}}}$$

where *A*_c_ is the contact area between the cell and the confining cantilever, *r*_c_ the corresponding radius and *P* the pressure across the plasma membrane. Using Pappus’ second centroid theorem, the volume *V* of the confined cell with circular side profiles of radius *r*

6 $$r = \frac{h}{2}$$

can be calculated as

7 $$V = \pi hr_{\rm{c}}^2 + 2\pi \left( {{r_{\rm{c}}} + \frac{{4r}}{{3\pi }}} \right) \times \pi \frac{{{r^2}}}{2}.$$

### Quantification of cortical myosin II and actin localization

HeLa Kyoto cells expressing MYH9-EGFP and Lifeact-mCherry, MYH9-EGFP or Actin-EGFP were prepared for experiments in glass bottom dishes, chemically arrested in mitosis in the presence of 2 µM STC and mounted on an inverted light microscope (AxioObserver.Z1, Zeiss) equipped with a laser scanning microscope (LSM 700, Zeiss) using a dish heater (JPK Instruments) at a constant temperature of 37 °C on a manual or automated precision stage (JPK Instruments). Imaging was carried out using a ×63 water-immersion objective (LCI Plan-Neofluar 63x/1.3 Imm Corr DIC M27) and ZEN acquisition software (Zeiss). Using modified (‘wedged’) cantilevers compensating for the 10° tilt intrinsic to our AFM setup and thus preventing cells from sliding towards the cantilever base^5, 19^, the cantilever was brought into contact with the glass surface close to the cell of interest, retracted by at least 22 µm, positioned over the cell and subsequently lowered with a constant speed of 0.5 µm s^−1^ to a predefined set height (10 or 12 µm). Images at the cellular midplane were recorded and cortical enrichment of myosin II and actin were analyzed by comparing the average fluorescence intensity of MYH9-EGFP, Lifeact-mCherry or Actin-EGFP at the cell edge to that in the cytoplasm using a custom Igor image analysis macro (Igor Pro)^11^.

### AFM-based height confinement of single interphase cells

HeLa Kyoto cells expressing H2B-EGFP and mCherry-CAAX were prepared for experiments in glass bottom dishes and chemically arrested in mitosis in presence of 2 µM STC as described above. Using modified cantilevers compensating for the 10° tilt intrinsic to the AFM setup, mitotic arrested cells were confined to a set height of typically 12 µm and images recorded as described above. Upon measuring a set of mitotic cells, the microscopy medium was removed from the petri dish, 200 µl of DMEM (high glucose, GlutaMAX supplement, pyruvate, Life Technologies) containing 10% (vol/vol) 10× trypsin (Trypsin (2.5%), no phenol red, no EDTA, Life Technologies) were added and cells incubated for 15–20 min. Then, microscopy medium containing 0.1% FBS was added to a total volume of 2 ml. Subsequently, rounded interphase cells were selected based on EGFP fluorescence showing an intact nucleus, and were confined to a set height of 10 µm. Images were recorded as described above for mitotic cells. Confinement experiments on interphase cells were typically started around 15 min and carried out within 90 min after addition of microscopy medium and dilution of trypsin.

### AFM-based trans-mitotic confinement of single cells

HeLa Kyoto cells expressing H2B-EGFP and mCherry-CAAX or H2B-mCherry and MYH9-EGFP were prepared for experiments as described above. Cells in late prophase (before nuclear envelope breakdown) were identified based on EGFP (H2B-EGFP) or mCherry (H2B-mCherry) fluorescence, and a modified cantilever compensating for the 10° tilt intrinsic to the AFM setup and thus preventing cells from sliding towards the cantilever base^5, 19^ was positioned at a predefined set height (typically 8 or 10 µm) above the cell. Images at the cellular midplane were recorded at a set time interval (usually 2 min). Care was taken to continuously adjust the focus to the cellular midplane as the cell rounded against the cantilever, and the corresponding resultant force trace was recorded. After chromosome segregation, the cell was released from confinement and the glass dish surface was probed with the cantilever to quantify potential time-dependent drift in cantilever height. For experiments where cortical myosin II localization was analyzed, images were processed and cortical localization values determined using the custom Igor image analysis macro (Igor Pro) as described above^11^.

### Detailed measurement of mitotic cell mechanics

For the detailed force/pressure measurement described in Fig. 5 and Supplementary Figs. 9–10, the tipless cantilever was first set at 14 µm height and placed over a metaphase cell to record rounding force. The cantilever was then brought down to 8 µm height at 0.1 µm s^−1^, to measure equilibrium force. The maximal cross-section area of the cell was used to calculate cell pressure and volume, as described above. In every experiment, MYH9 RNAi was employed as a control for the decreased pressure.

### Light microscopy and image analysis

Immunofluorescence analysis was performed as following^24^: HeLa cells were fixed in 3% paraformaldehyde in phosphate-buffered saline (PBS) supplemented with 5 mM EGTA and 1 mM MgCl_2_, washed with PBS with 30 mM glycine, permeabilized with 0.1% Triton X-100, and blocked with 0.2% fish skin gelatin (Sigma). Goat anti-EGFP^24^ (used at 1: 2000 dilution), mouse anti-tubulin (DM1A, Sigma, 1: 1000) and AlexaFluor-tagged secondary antibodies (A-11055 and A-21203, Thermo Fisher Scientific, 1: 500) were used for immunostaining. DNA was stained with 100 ng ml^−1^ DAPI (Calbiochem). Images were taken on a DeltaVision system (Applied Precision) equipped with a CoolSnap HQ CCD camera (Roper Scientific), deconvolved and maximally projected.

For imaging of ER and total mitochondria, ER Tracker Red (E34250, Thermo Fisher Scientific, 100 nM) and MitoTracker Red CMXRos (M7512, Thermo, 150 nM) were used, respectively. They were imaged on the DeltaVision system equipped with a CO_2_ supply, deconvolved and maximally projected.

### Chemicals

Glycolic acid (15451, ACROS Organics) neutralized with NaOH to pH 7.4 to prepare glycolate, sodium D-lactate (71716, Sigma) and sodium L-lactate (L7022, Sigma) were used.

### Immunoblotting

MEF cells were lysed in a lysis buffer (50 mM HEPES pH 7.5, 150 mM KCl, 1 mM MgCl_2_, 10% glycerol, 0.1% NP-40) with a protease inhibitor cocktail (Complete, Roche) and mixed with 2× SDS sample buffer (125 mM Tris-HCl (pH 6.8), 4% SDS, 20% glycerol, 5% 2-mercaptoethanol and 0.01% bromophenolblue). HeLa cells were lysed using 2× SDS sample buffer. Cells were boiled for five minutes, resolved in SDS-PAGE, and transferred onto a nitrocellulose membrane. In immunoblotting, the following primary antibodies were used: mouse DJ-1 (HPA004190, Sigma, diluted by 1: 250); alpha-tubulin (DM1A, Sigma, 1: 2000); GAPDH (#2118, Cell Signaling Technology, 1: 1000), and custom-made rabbit polyclonal anti-FAM134A antibody (C16039 and C16045, raised in two rabbits against synthetic peptide CDAPPLGPDIHSLVQS coupled to KLH, harvested and affinity-purified at the Antibody Facility of MPI-CBG in Dresden, 1: 2000). Horseradish peroxidase-conjugated anti-IgG antibodies (#170-6515 and #170-6516, Bio-rad, 1: 2000) were used for the secondary antibodies. Chemiluminescence by ECL reagent (Amersham ECL Western Blotting Detection Reagent, GE Healthcare Life Sciences) was detected on a Hyperfilm (GE healthcare) or X-ray film (Super RX, Fujifilm).

### Cell fractionation

Cell fractionation into cytoplasmic, membrane, nuclear soluble, chromatin-bound and cytoskeletal protein fractions was carried out using a fractionation kit (#78840, Thermo Fisher Scientific) following the manufacturer instructions. The equivalent of 50,000 cells was used for immunoblotting.

### Statistics and data representation

Statistical differences between the conditions of the AFM measurements were determined by a Student’s *t*-test for the screen or a nonparametric unpaired Mann-Whitney U test for the post-screen experiments (Figs. 3–5 and Supplementary Figs. 1, 5-7, 9–10). In both cases, *p*-values ≤ 0.05 were considered statistically significant. Statistical analysis and graphs were made on Prism software version 5 (GraphPad Inc.).

### Data availability

The authors declare that all data supporting the findings of this study are available within the article and its Supplementary Information files. Extra data are available from the corresponding authors upon request.

## Electronic supplementary material

Supplementary Information

Description of Additional Supplementary Files

Supplementary Data 1

Supplementary Data 2

Supplementary Data 3

Supplementary Data 4

Supplementary Movie 1

Supplementary Movie 2

## References

[1] Thery M, Bornens M (2008). Get round and stiff for mitosis. HFSP J.

[2] Ramkumar N, Baum B (2016). Coupling changes in cell shape to chromosome segregation. Nat. Rev. Mol. Cell Biol..

[3] Lancaster OM (2013). Mitotic rounding alters cell geometry to ensure efficient bipolar spindle formation. Dev. Cell.

[4] Cadart C, Zlotek-Zlotkiewicz E, Le Berre M, Piel M, Matthews HK (2014). Exploring the function of cell shape and size during mitosis. Dev. Cell.

[5] Cattin CJ (2015). Mechanical control of mitotic progression in single animal cells. Proc. Natl Acad. Sci. USA.

[6] Kondo T, Hayashi S (2013). Mitotic cell rounding accelerates epithelial invagination. Nature..

[7] Weber IP (2014). Mitotic position and morphology of committed precursor cells in the zebrafish retina adapt to architectural changes upon tissue maturation. Cell Rep..

[8] Hoijman E, Rubbini D, Colombelli J, Alsina B (2015). Mitotic cell rounding and epithelial thinning regulate lumen growth and shape. Nat. Commun..

[9] Nakajima Y, Meyer EJ, Kroesen A, McKinney SA, Gibson MC (2013). Epithelial junctions maintain tissue architecture by directing planar spindle orientation. Nature.

[10] Stewart MP (2011). Hydrostatic pressure and the actomyosin cortex drive mitotic cell rounding. Nature.

[11] Ramanathan SP (2015). Cdk1-dependent mitotic enrichment of cortical myosin II promotes cell rounding against confinement. Nat. Cell Biol..

[12] Sorce B (2015). Mitotic cells contract actomyosin cortex and generate pressure to round against or escape epithelial confinement. Nat. Commun..

[13] Nurse P (2002). Cyclin dependent kinases and cell cycle control (nobel lecture). Chembiochem.

[14] Clark AG, Paluch E (2011). Mechanics and regulation of cell shape during the cell cycle. Results Probl. Cell Differ..

[15] Salbreux G, Charras G, Paluch E (2012). Actin cortex mechanics and cellular morphogenesis. Trends Cell Biol..

[16] Fischer-Friedrich E, Hyman AA, Julicher F, Muller DJ, Helenius J (2014). Quantification of surface tension and internal pressure generated by single mitotic cells. Sci. Rep..

[17] Matthews HK (2012). Changes in Ect2 localization couple actomyosin-dependent cell shape changes to mitotic progression. Dev. Cell.

[18] Stewart MP, Toyoda Y, Hyman AA, Muller DJ (2012). Tracking mechanics and volume of globular cells with atomic force microscopy using a constant-height clamp. Nat. Protoc..

[19] Stewart MP (2013). Wedged AFM-cantilevers for parallel plate cell mechanics. Methods.

[20] Skoufias DA (2006). S-trityl-L-cysteine is a reversible, tight binding inhibitor of the human kinesin Eg5 that specifically blocks mitotic progression. J. Biol. Chem..

[21] Kittler R (2007). Genome-wide resources of endoribonuclease-prepared short interfering RNAs for specific loss-of-function studies. Nat. Methods.

[22] Myers JW (2006). Minimizing off-target effects by using diced siRNAs for RNA interference. J RNAi Gene Silencing.

[23] Toyoda Y, Stewart MP, Hyman AA, Muller DJ (2011). Atomic force microscopy to study mechanics of living mitotic Mammalian cells. Jpn. J. Appl. Phys..

[24] Hutchins JR (2010). Systematic analysis of human protein complexes identifies chromosome segregation proteins. Science.

[25] Safran M (2010). GeneCards version 3: the human gene integrator. Database (Oxford).

[26] Poser I (2008). BAC TransgeneOmics: a high-throughput method for exploration of protein function in mammals. Nat. Methods.

[27] Fededa JP, Gerlich DW (2012). Molecular control of animal cell cytokinesis. Nat. Cell Biol..

[28] Laporte D, Ojkic N, Vavylonis D, Wu JQ (2012). Alpha-actinin and fimbrin cooperate with myosin II to organize actomyosin bundles during contractile-ring assembly. Mol. Biol. Cell.

[29] Mukhina S, Wang YL, Murata-Hori M (2007). Alpha-actinin is required for tightly regulated remodeling of the actin cortical network during cytokinesis. Dev. Cell.

[30] Courson DS, Rock RS (2010). Actin cross-link assembly and disassembly mechanics for alpha-Actinin and fascin. J. Biol. Chem..

[31] Maddox AS, Burridge K (2003). RhoA is required for cortical retraction and rigidity during mitotic cell rounding. J. Cell Biol..

[32] Musacchio A (2015). The molecular biology of spindle assembly checkpoint signaling dynamics. Curr. Biol..

[33] Kittler R (2007). Genome-scale RNAi profiling of cell division in human tissue culture cells. Nat. Cell Biol..

[34] Neumann B (2010). Phenotypic profiling of the human genome by time-lapse microscopy reveals cell division genes. Nature..

[35] Bonifati V (2003). Mutations in the DJ-1 gene associated with autosomal recessive early-onset parkinsonism. Science.

[36] Fischer-Friedrich E (2016). Rheology of the active cell cortex in mitosis. Biophys. J..

[37] Berryman M, Bruno J, Price J, Edwards JC (2004). CLIC-5A functions as a chloride channel in vitro and associates with the cortical actin cytoskeleton in vitro and in vivo. J. Biol. Chem..

[38] Singh H, Cousin MA, Ashley RH (2007). Functional reconstitution of mammalian ‘chloride intracellular channels’ CLIC1, CLIC4 and CLIC5 reveals differential regulation by cytoskeletal actin. FEBS J..

[39] Wegner B (2010). CLIC5A, a component of the ezrin-podocalyxin complex in glomeruli, is a determinant of podocyte integrity. Am. J. Physiol. Renal. Physiol..

[40] Gagnon LH (2006). The chloride intracellular channel protein CLIC5 is expressed at high levels in hair cell stereocilia and is essential for normal inner ear function. J. Neurosci..

[41] Pham TT (2010). DJ-1-deficient mice show less TH-positive neurons in the ventral tegmental area and exhibit non-motoric behavioural impairments. Genes Brain Behav..

[42] Hasim S (2014). A glutathione-independent glyoxalase of the DJ-1 superfamily plays an important role in managing metabolically generated methylglyoxal in Candida albicans. J. Biol. Chem..

[43] Nagakubo D (1997). DJ-1, a novel oncogene which transforms mouse NIH3T3 cells in cooperation with ras. Biochem. Biophys. Res. Commun..

[44] Lee JY (2012). Human DJ-1 and its homologs are novel glyoxalases. Hum. Mol. Genet..

[45] Clark IE (2006). Drosophila pink1 is required for mitochondrial function and interacts genetically with parkin. Nature.

[46] Park J (2006). Mitochondrial dysfunction in Drosophila PINK1 mutants is complemented by parkin. Nature.

[47] Toyoda Y (2014). Products of the Parkinson’s disease-related glyoxalase DJ-1, D-lactate and glycolate, support mitochondrial membrane potential and neuronal survival. Biol Open.

[48] Fuchs F (2010). Clustering phenotype populations by genome-wide RNAi and multiparametric imaging. Mol. Syst. Biol..

[49] Rohn JL (2011). Comparative RNAi screening identifies a conserved core metazoan actinome by phenotype. J. Cell. Biol..

[50] Kitada T, Tong Y, Gautier CA, Shen J (2009). Absence of nigral degeneration in aged parkin/DJ-1/PINK1 triple knockout mice. J. Neurochem..

[51] Stewart MP, Toyoda Y, Hyman AA, Muller DJ (2011). Force probing cell shape changes to molecular resolution. Trends. Biochem. Sci..

[52] Rosa A, Vlassaks E, Pichaud F, Baum B (2015). Ect2/Pbl acts via Rho and polarity proteins to direct the assembly of an isotropic actomyosin cortex upon mitotic entry. Dev. Cell.

[53] Boucrot E, Howes MT, Kirchhausen T, Parton RG (2011). Redistribution of caveolae during mitosis. J. Cell Sci..

[54] Ozlu N (2015). Quantitative comparison of a human cancer cell surface proteome between interphase and mitosis. EMBO J..

[55] Kim RH (2005). Hypersensitivity of DJ-1-deficient mice to 1-methyl-4-phenyl-1,2,3,6-tetrahydropyrindine (MPTP) and oxidative stress. Proc. Natl Acad. Sci. USA.

[56] Rabbani N, Thornalley PJ (2011). Glyoxalase in diabetes, obesity and related disorders. Semin. Cell Dev. Biol..

[57] Flick MJ, Konieczny SF (2002). Identification of putative mammalian D-lactate dehydrogenase enzymes. Biochem. Biophys. Res. Commun..

[58] Byun S (2013). Characterizing deformability and surface friction of cancer cells. Proc. Natl Acad. Sci. USA.

[59] Nyberg KD (2016). The physical origins of transit time measurements for rapid, single cell mechanotyping. Lab Chip..

[60] Augustsson P, Karlsen JT, Su HW, Bruus H, Voldman J (2016). Iso-acoustic focusing of cells for size-insensitive acousto-mechanical phenotyping. Nat. Commun..

[61] Otto O (2015). Real-time deformability cytometry: on-the-fly cell mechanical phenotyping. Nat. Methods.

[62] Gossett DR (2012). Hydrodynamic stretching of single cells for large population mechanical phenotyping. Proc. Natl Acad. Sci. USA.

[63] Darling EM, Di Carlo D (2015). High-throughput assessment of cellular mechanical properties. Annu. Rev. Biomed. Eng..

[64] Muller DJ, Helenius J, Alsteens D, Dufrene YF (2009). Force probing surfaces of living cells to molecular resolution. Nat. Chem. Biol..

[65] Garcia R, Herruzo ET (2012). The emergence of multifrequency force microscopy. Nat. Nanotechnol..

[66] Maliga Z (2013). A genomic toolkit to investigate kinesin and myosin motor function in cells. Nat. Cell Biol..

[67] UniProt C (2015). UniProt: a hub for protein information. Nucleic Acids Res..

[68] Kanehisa M, Goto S (2000). KEGG: kyoto encyclopedia of genes and genomes. Nucleic Acids Res..

[69] Hutter JL, Bechhoefer J (1993). Calibration of atomic-force microscope tips. Rev. Sci. Instrum..

[70] Surendranath V, Theis M, Habermann BH, Buchholz F (2013). Designing efficient and specific endoribonuclease-prepared siRNAs. Methods Mol. Biol..

