# Supplementary material for: Genome-scale single-cell mechanical phenotyping reveals disease-related genes involved in mitotic rounding
Source: Nat Commun. 2017 Nov 2;8:1266. doi: 10.1038/s41467-017-01147-6 (PMC5668354; doi:10.1038/s41467-017-01147-6)
Supplement: Supplementary file 1 — Supplementary Information [file 41467_2017_1147_MOESM1_ESM.pdf]

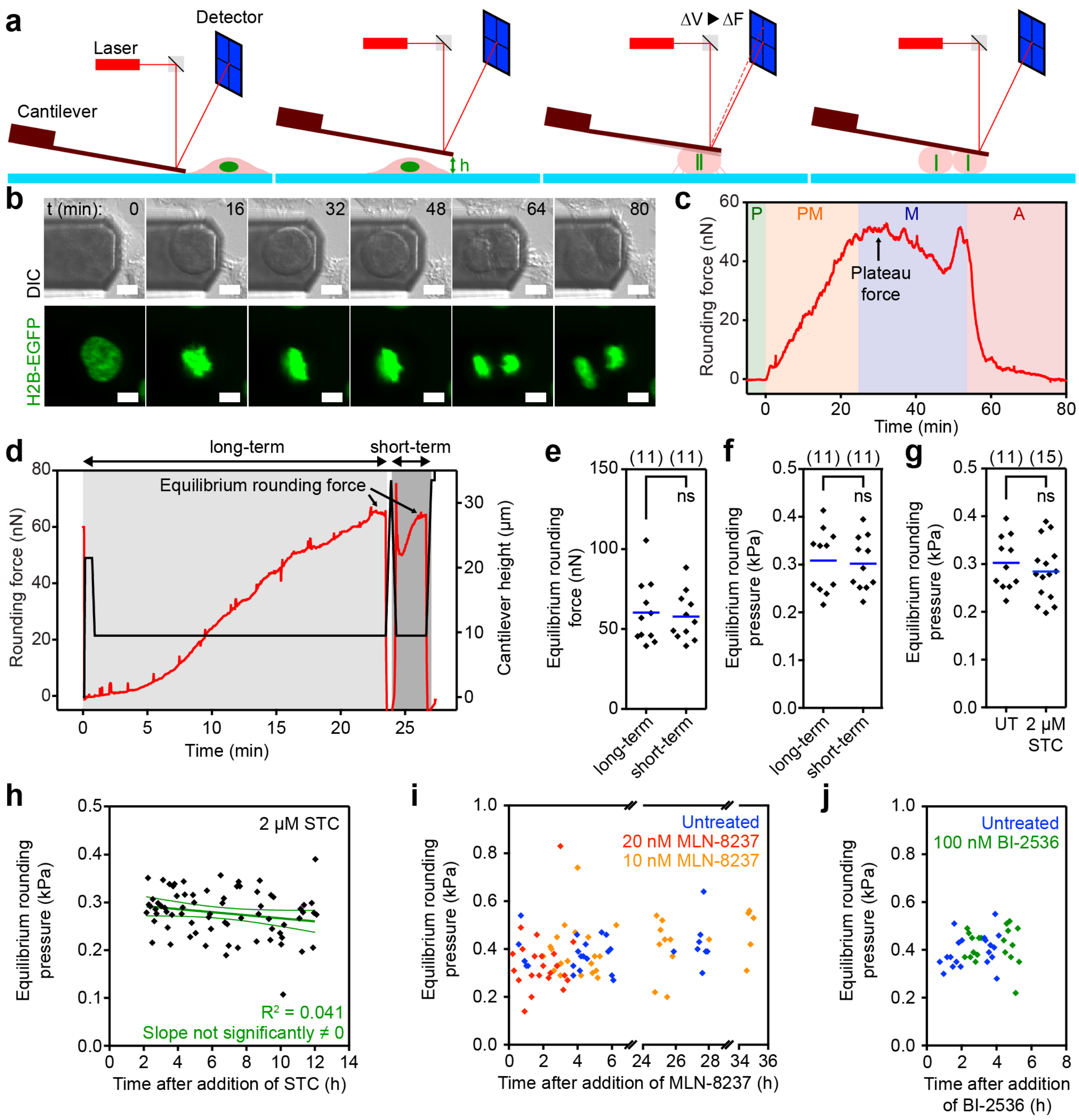

Supplementary Figure 1. A short-term height confinement assay in the presence of STC is applicable to measure equilibrium rounding force and pressure of mitotic cells. (**a**) Depiction the AFM-based microcantilever assay to characterize the mechanical properties of a confined cell progressing through mitosis. (**b-c**) Differential interference contrast (DIC) (**b,** top), fluorescence images (**b**, bottom) and rounding force (**c**) generated by a H2B-EGFP expressing HeLa cell during mitosis. As the cell rounds against the cantilever and deflects it upwards, the recorded force acting on the cantilever increases until a plateau force is reached. A distinct peak in the force trace marks the onset of anaphase. $t = 0$, onset of nuclear envelope breakdown. h, cantilever height above substrate. Colored background in (**c**) indicates mitotic phases: P, prophase; PM, prometaphase; M, metaphase; A, anaphase. (**d**) Example force trace (red) of a mitotic HeLa cell rounding against a confining cantilever held at a fixed height (10 μm, black trace). $t = 0$, start of the measurement at the transition of prophase to prometaphase. Once the cell reaches an equilibrium rounding force in metaphase, the cantilever is withdrawn and subsequently height confinement (10 μm) is re-established. Equilibrium rounding forces of long-term (light grey background) and short-term (dark grey) measurements are recorded. (**e-f**) Equilibrium rounding force (**e**) and pressure (**f**) for cells submitted to the assay exemplified in (**d**). (**g**) Equilibrium

rounding pressure from short-term height confinement of untreated (UT) and chemically arrested (S-trityl-L-cysteine, STC) confined mitotic cells submitted to the assay exemplified in (**d**). (**h**) Equilibrium rounding pressure of STC-arrested mitotic cells *versus* time with best-fit (thick green line) and 95% confidence intervals (thin green lines) of a linear regression. (**i**) Equilibrium rounding pressure of mitotic cells treated with aurora kinase A inhibitor MLN-8237[1] and untreated mitotic cells *versus* time. (**j**) Equilibrium rounding pressure of mitotic cells treated with polo-like kinase 1 (PLK1) inhibitor BI-2536[2,3] and untreated mitotic cells *versus* time. The inhibitors were added at time zero. Each diamond represents one cell. '(n)', number of cells measured. Blue bars, mean. Statistical significance was determined by Mann-Whitney U test (ns, $p > 0.05$). Scale bars, 10 μm.

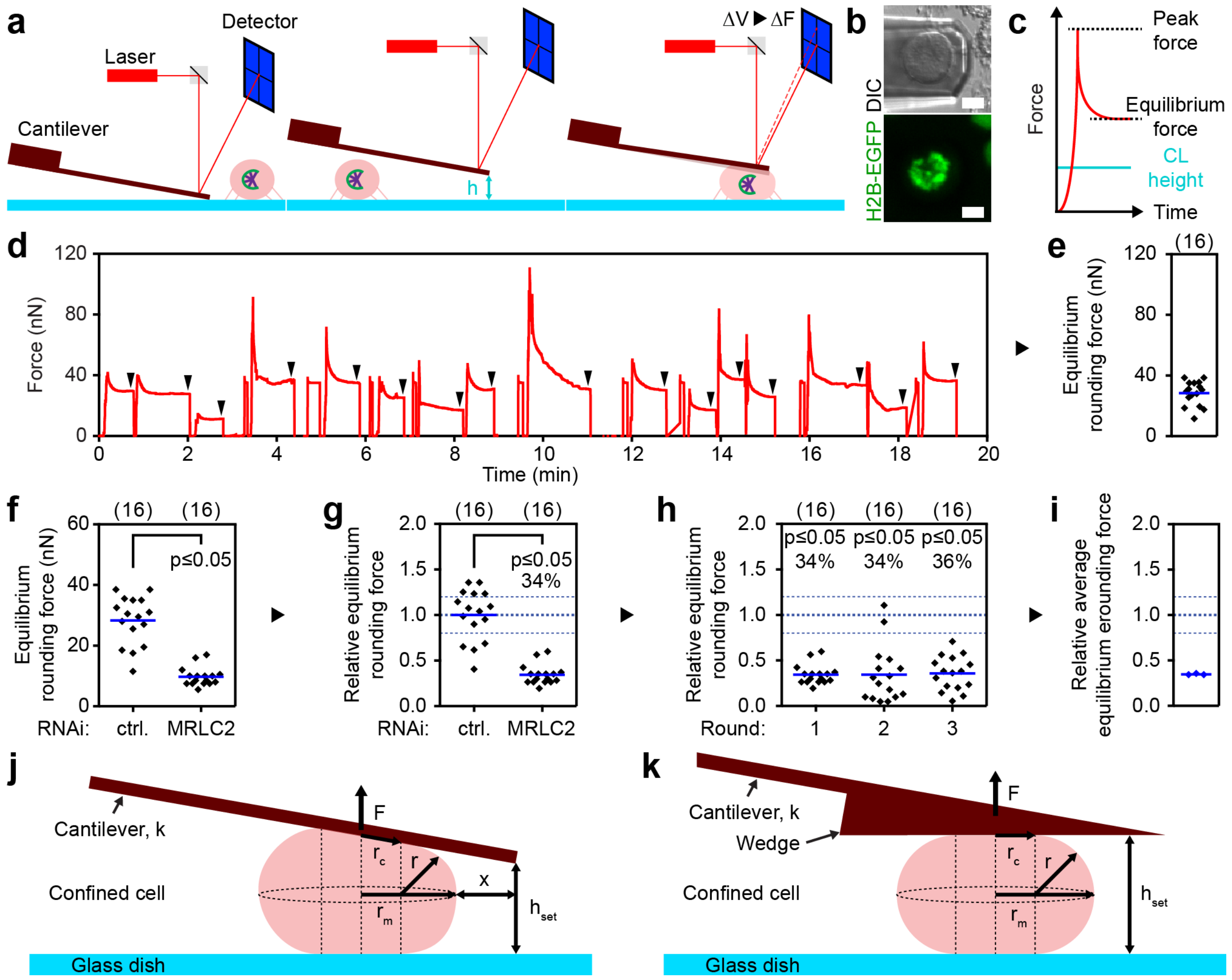

Supplementary Figure 2. Assay depiction and screen workflow. (**a-c**) Depiction (**a**), differential interference contrast (DIC) as well as fluorescence images (**b**) and example force trace (**c**) of a H2B-EGFP expressing HeLa cell in the confinement assay used for screening. A tipless cantilever is brought into contact with the glass surface close to a STC-arrested mitotic cell (**a**, left), retracted by 14 μm (middle) and positioned over the cell (right). Images of the confined cell are taken (**b**) and the cantilever deflection upon contact with the cell (i.e. the rounding force) is recorded over time as exemplified in (**c**). h, cantilever (CL) height above substrate. For details see Methods. (**d-e**) Example force profile of consecutive measurements on 16 different cells (**d**). Arrowheads denote the equilibrium rounding forces per cell, which are plotted in (**e**). (**f-i**) Exemplified analysis workflow. (**f**) Equilibrium rounding forces of STC-arrested mitotic control cells (ctrl., esiRNA targeting firefly luciferase,) and cells treated with esiRNA targeting the myosin regulatory light chain MRLC2/MYL12B. (**g**) Equilibrium rounding force from (**f**), normalized to the average rounding force of control cells. (**h**) Relative equilibrium rounding force of MRLC2-depleted cells from three rounds. Each black diamond corresponds to the equilibrium rounding force of one mitotic cell. '(*n*)', number of cells measured. (**i**) Relative average equilibrium rounding forces from (**h**). Blue diamonds in (**i**) correspond to relative averages (blue bars) in (**h**). Blue bars, mean. Thick (upper and lower thin) blue dotted lines denote 100% (120% and 80%) of the average of control cells. (**j-k**) Depicted geometry of a rounded cell confined by a non-wedged cantilever (**j**) and a wedged cantilever (compensating for the 10 ° mounting angle[4,5]) (**k**) with measures, based on a model cell with cortical shell and liquid core[6]. k, cantilever spring constant; F, measured force; r_c, radius of cell-cantilever contact area; r_m, radius of cellular midplane cross section; r, radius of circular side profile; x, distance from cantilever tip to cell edge; h_{set}, preset cantilever height. For corresponding formulas see Methods. Statistically significant differences are denoted by Student's t-tests ($p \leq 0.05$). Percentage values denote difference of average to average of control cells. Scale bars, 10 μm.

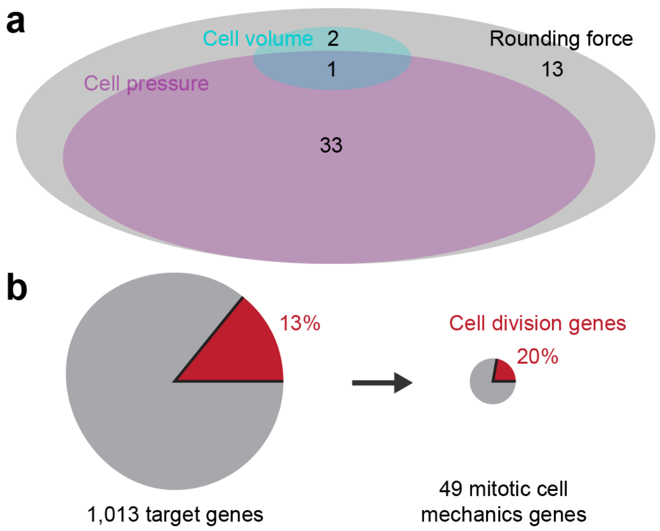

Supplementary Figure 3. Classification of the mitotic cell mechanics genes. (**a**) Classification of the 49 mitotic cell mechanics genes identified in the RNAi-based screen described in **Fig. 1**. The number of genes that affected intracellular pressure (magenta), cell volume (turquoise), and rounding force only (grey) upon protein depletion by RNAi is shown. Relative areas of ovals represent percentage of genes per category. (**b**) Comparison of enrichment of genes required for cell division. The percentages of cell division genes (red) in all target genes (left) and in the 49 mitotic cell mechanics genes (right) are shown. Cell division genes are genes found to be required for mitotic progression, chromosome segregation, and/or cell division in earlier genome-scale RNAi screens using esiRNA or siRNA[7,8].

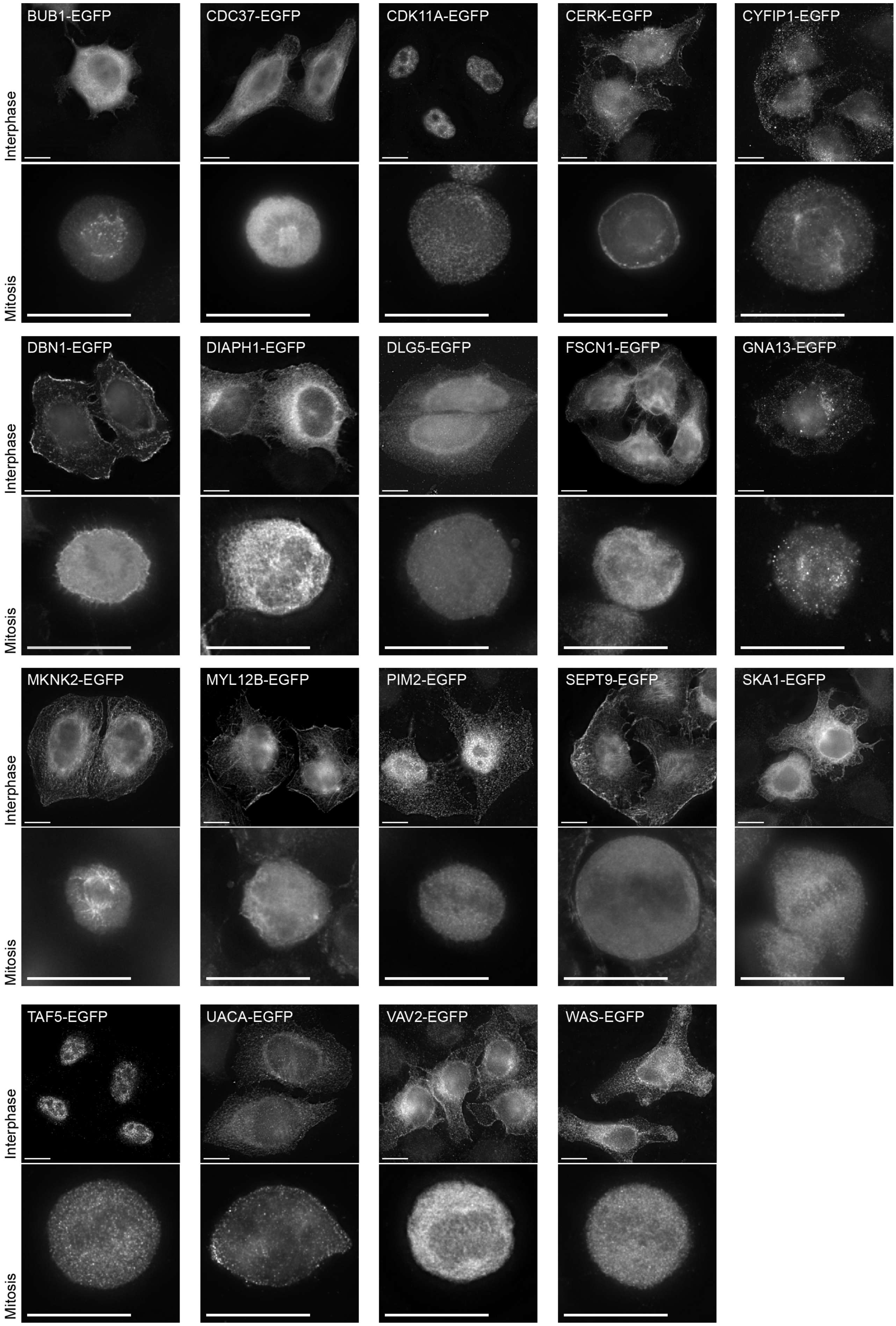
BUB1-EGFP
CDC37-EGFP
CDK11A-EGFP
CERK-EGFP
CYFIP1-EGFP
DBN1-EGFP
DIAPH1-EGFP
DLG5-EGFP
FSCN1-EGFP
GNA13-EGFP
MKNK2-EGFP
MYL12B-EGFP
PIM2-EGFP
SEPT9-EGFP
SKA1-EGFP
TAF5-EGFP
UACA-EGFP
VAV2-EGFP
WAS-EGFP
Interphase
Mitosis

Supplementary Figure 4. Subcellular localization of further selected mitotic cell mechanics proteins. Immunofluorescence images of fixed HeLa interphase (top) and mitotic (bottom) cells expressing 19 selected EGFP-tagged proteins, which our screen identified to cause mechanical phenotypes of mitotic cells when downregulated by RNAi (**Fig. 1**). The proteins BUB1, CDC37, CDK11A, CERK, CYFIP1, DBN1, DIAPH1, DLG5, FSCN1, GNA13, MKNK2, MYL12B, PIM2, SEPT9, SKA1, TAF5, UACA, VAV2, and WAS were tagged using the BAC TransgeneOmics technology[9]. Stable cell lines were fixed and stained with a goat anti-GFP antibody/Alexa-488. Scale bars, 10 μm.

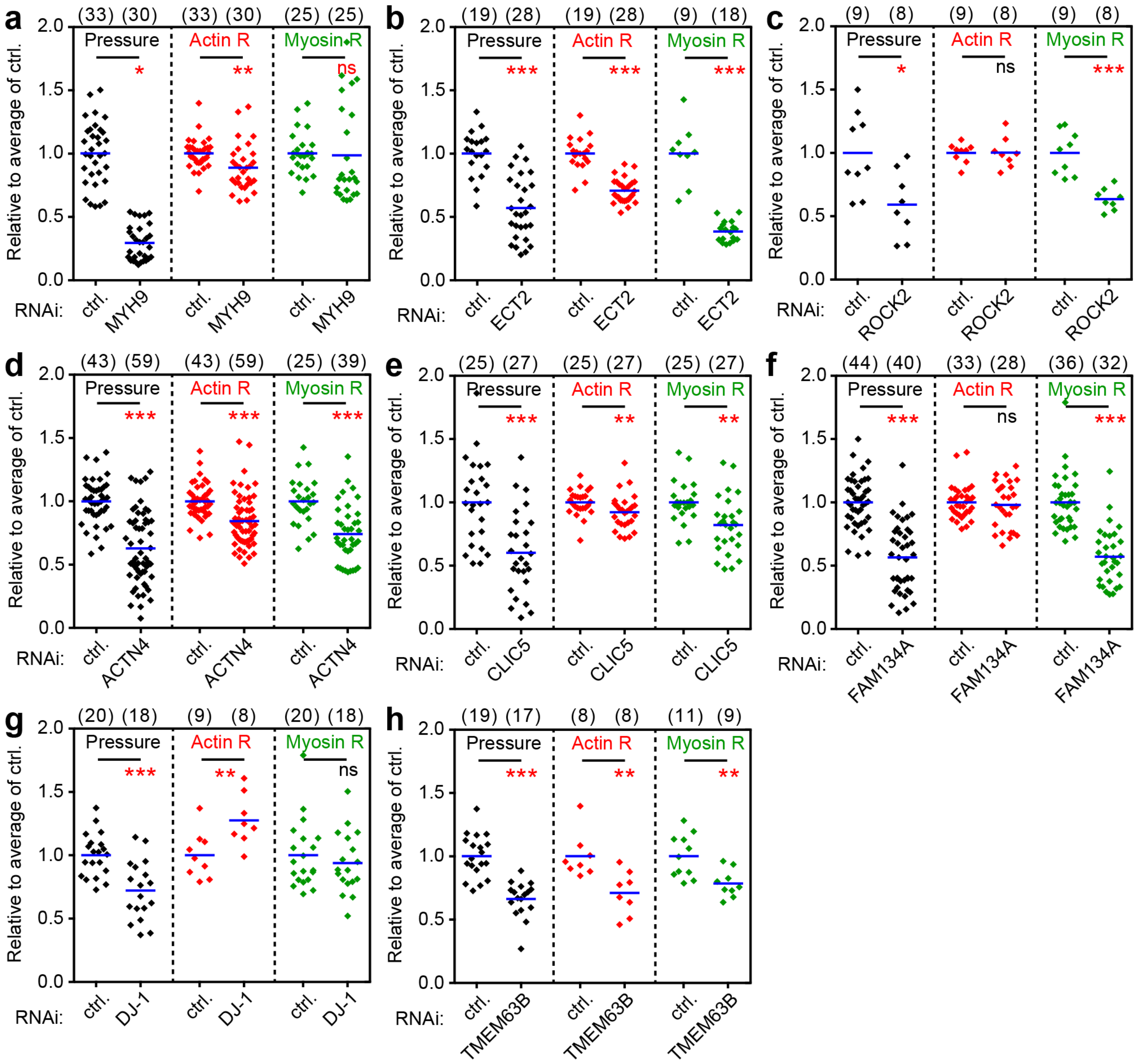

Supplementary Figure 5. Influence of RNAi-based knockdown of selected mitotic cell mechanics genes on the cortical enrichment of F-actin and myosin II. (**a-h**) Relative equilibrium rounding pressure (left), relative cortex to cytoplasm ratio for F-actin (Actin R, middle) and myosin II (Myosin R, right) for confined STC-arrested mitotic Actin-EGFP, MYH9-EGFP or Lifeact-mCherry and MYH9-EGFP expressing HeLa cells depleted of MYH9 (**a**), ECT2 (**b**), ROCK2 (**c**), ACTN4 (**d**), CLIC5 (**e**), FAM134A (**f**), DJ-1 (**g**) or TMEM63B (**h**) by RNAi. Control (ctrl.) cells were RNAi treated with negative control dsRNA (AllStars siRNA or firefly luciferase esiRNA). Values relative to respective control data average. Each diamond corresponds to one cell. '(*n*)' denotes number of cells measured. Blue bars, mean. Statistical significance was determined by Mann-Whitney U test (ns, $p > 0.05$; *, $p \leq 0.05$; **, $p < 0.01$; ***, $p < 0.001$).

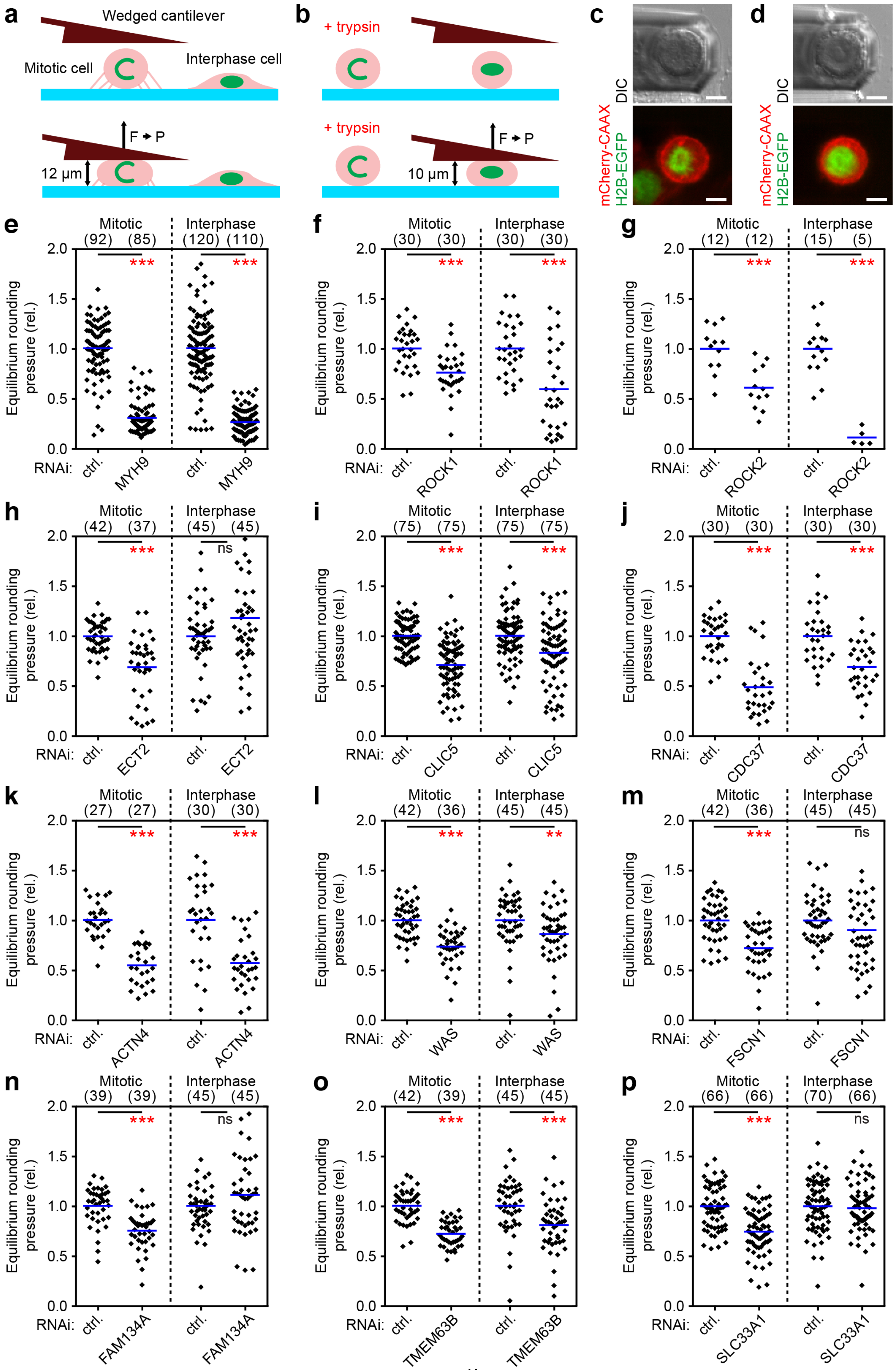
a
Wedged cantilever
Mitotic cell
Interphase cell
F → P
12 µm
b
+ trypsin
+ trypsin
F → P
10 µm
c
DIC
mCherry-CAAX
H2B-EGFP
d
DIC
mCherry-CAAX
H2B-EGFP
e
Mitotic
Interphase
(92) (85) (120) (110)
***
***
Equilibrium rounding pressure (rel.)
RNAi: ctrl. MYH9 ctrl. MYH9
f
Mitotic
Interphase
(30) (30) (30) (30)
***
***
Equilibrium rounding pressure (rel.)
RNAi: ctrl. ROCK1 ctrl. ROCK1
g
Mitotic
Interphase
(12) (12) (15) (5)
***
***
Equilibrium rounding pressure (rel.)
RNAi: ctrl. ROCK2 ctrl. ROCK2
h
Mitotic
Interphase
(42) (37) (45) (45)
***
ns
Equilibrium rounding pressure (rel.)
RNAi: ctrl. ECT2 ctrl. ECT2
i
Mitotic
Interphase
(75) (75) (75) (75)
***
***
Equilibrium rounding pressure (rel.)
RNAi: ctrl. CLIC5 ctrl. CLIC5
j
Mitotic
Interphase
(30) (30) (30) (30)
***
***
Equilibrium rounding pressure (rel.)
RNAi: ctrl. CDC37 ctrl. CDC37
k
Mitotic
Interphase
(27) (27) (30) (30)
***
***
Equilibrium rounding pressure (rel.)
RNAi: ctrl. ACTN4 ctrl. ACTN4
l
Mitotic
Interphase
(42) (36) (45) (45)
***
**
Equilibrium rounding pressure (rel.)
RNAi: ctrl. WAS ctrl. WAS
m
Mitotic
Interphase
(42) (36) (45) (45)
***
ns
Equilibrium rounding pressure (rel.)
RNAi: ctrl. FSCN1 ctrl. FSCN1
n
Mitotic
Interphase
(39) (39) (45) (45)
***
ns
Equilibrium rounding pressure (rel.)
RNAi: ctrl. FAM134A ctrl. FAM134A
o
Mitotic
Interphase
(42) (39) (45) (45)
***
***
Equilibrium rounding pressure (rel.)
RNAi: ctrl. TMEM63B ctrl. TMEM63B
p
Mitotic
Interphase
(66) (66) (70) (66)
***
ns
Equilibrium rounding pressure (rel.)
RNAi: ctrl. SLC33A1 ctrl. SLC33A1
0.0
0.5
1.0
1.5
2.0

Supplementary Figure 6. Analysis of equilibrium rounding pressure in mitotic and interphase cells reveals a mitotic-specific effect for ECT2, FSCN1, FAM134A and SLC33A1. (**a-b**) Assay for evaluating mechanical response of mitotic and interphase cells. (**a**) First, the impaired rounding pressure phenotype was confirmed in STC-arrested mitotic cells. Mechanical response of RNAi-treated cells was confirmed using wedged microcantilevers. (**b**) Next, rounding pressure of trypsinized interphase cells was measured in the same culture dish (see Methods). P, rounding pressure; F, rounding force. (**c-d**) Typical DIC (top) as well as overlay of chromatin (H2B-EGFP) and cell membrane (mCherry-CAAX, bottom) images of confined STC-arrested mitotic (**c**) and interphase (**d**) cells. Arrested mitotic cells were identified by condensed chromosomes while interphase cells exhibit an intact nucleus. Scale bars, 10 μm. (**e-p**) Equilibrium rounding pressure of STC-arrested HeLa mitotic (left) and trypsin-treated interphase (right) cells depleted of MYH9 (**e**), ROCK1 (**f**), ROCK2 (**g**), ECT2 (**h**), CLIC5 (**i**), CDC37 (**j**), ACTN4 (**k**), WAS (**l**), FSCN1 (**m**), FAM134A (**n**), TMEM63B (**o**) or SCL33A1 (**p**) by RNAi. Control (ctrl.) cells RNAi treated with negative control (e)siRNA (AllStars siRNA or firefly luciferase esiRNA). Values relative to respective control data average. Each diamond corresponds to one cell. '(n)' denotes number of cells measured. Blue bars, mean. Statistical significance was determined by Mann-Whitney U test (ns, $p > 0.05$; *, $p \leq 0.05$; **, $p < 0.01$; ***, $p < 0.001$).

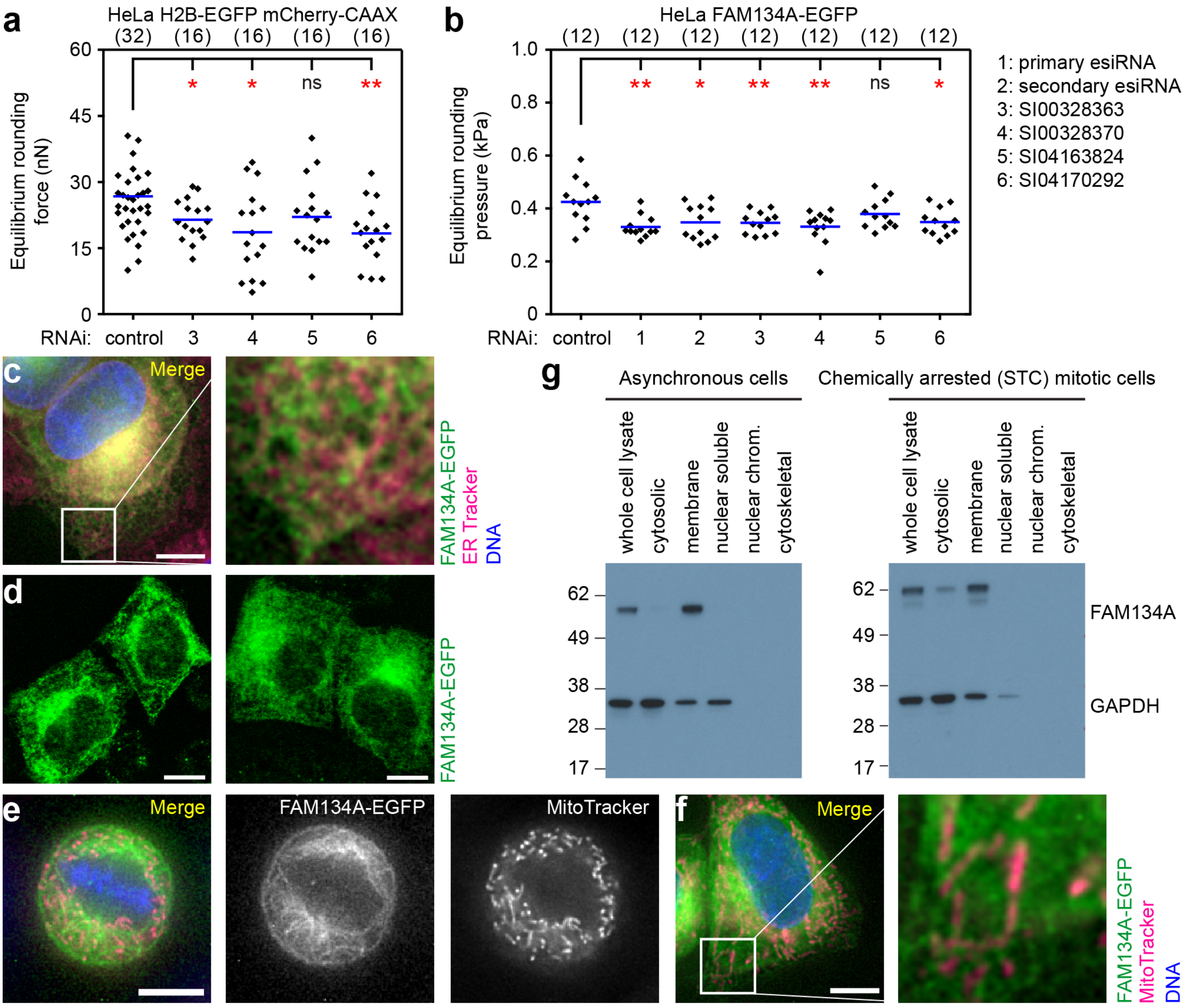

Supplementary Figure 7. An endoplasmic reticulum protein FAM134A is required for mitotic cell rounding pressure. (**a**) Equilibrium rounding force of STC-arrested H2B-EGFP and mCherry-CAAX expressing RNAi treated mitotic cells. Cells were transfected with the indicated siRNAs targeting FAM134A (see legend in (**b**)). Control, cells transfected with AllStars negative control siRNA. (**b**) Equilibrium rounding pressure of STC-arrested FAM134A-EGFP expressing RNAi treated mitotic cells. Cells were transfected with the indicated (e)siRNAs targeting FAM134A (see legend). Control cells transfected with AllStars negative control siRNA. (**c**) Predominantly perinuclear colocalization of FAM134A-EGFP (green) with the endoplasmic reticulum (ER) in an interphase cell. Blue, DNA; Magenta, ER Tracker, an ER marker. Yellow regions represent co-localization between FAM134A and ER. (**d**) Typical confocal images of FAM134A-EGFP in interphase cells. A meshwork-like signal with occasional cell surface localization was observed. (**e-f**) Comparison of FAM134A-EGFP (green) with MitoTracker (Magenta), a mitochondria marker. Shown are a typical mitotic cell (**e**) and a typical interphase cell (**f**). Blue, DNA. (**g**) Detection of endogenous FAM134A protein in fractionated cells. Lysates from asynchronous (left) and STC-arrested mitotic (right) HeLa cells were fractionated. Endogenous FAM134A protein was probed by immunoblotting. GAPDH serves as a loading control protein. For details about sample preparation and immunoblotting see Methods. Each diamond corresponds to one cell. '(*n*)' denotes number of cells measured. Blue bars, mean. Statistical significance was determined by Mann-Whitney U test (ns, $p > 0.05$; *, $p \leq 0.05$; **, $p < 0.01$). Scale bars, 10 μm.

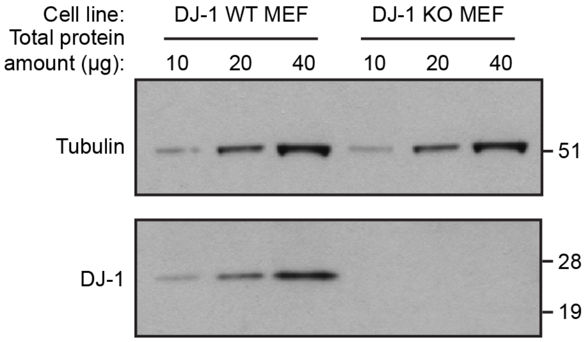

Supplementary Figure 8. Expression of DJ-1 protein in mouse embryonic fibroblasts. Immunoblot of DJ-1 using lysates from wild type (DJ-1 WT) and DJ-1 knockout (DJ-1 KO) mouse embryonic fibroblasts (MEFs). The total amount of protein loaded is stated above each lane. Tubulin serves as a loading control. For details about sample preparation and immunoblotting see Methods.

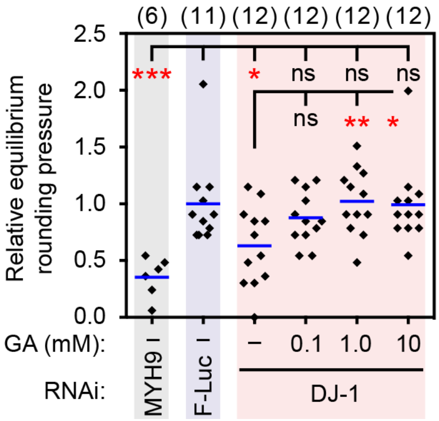

Supplementary Figure 9. Titration of the rescue effect of glycolate. Equilibrium rounding pressure of RNAi treated mitotic HeLa cells, incubated with the indicated concentration of glycolate (GA) for one hour. Values relative to firefly luciferase (F-Luc) control data average are shown. Each diamond corresponds to one cell. '(*n*)' denotes number of cells measured. Blue bars, mean. Statistical significance was determined by Mann-Whitney U test (ns, $p > 0.05$; *, $p \leq 0.05$; **, $p < 0.01$, ***, $p < 0.001$).

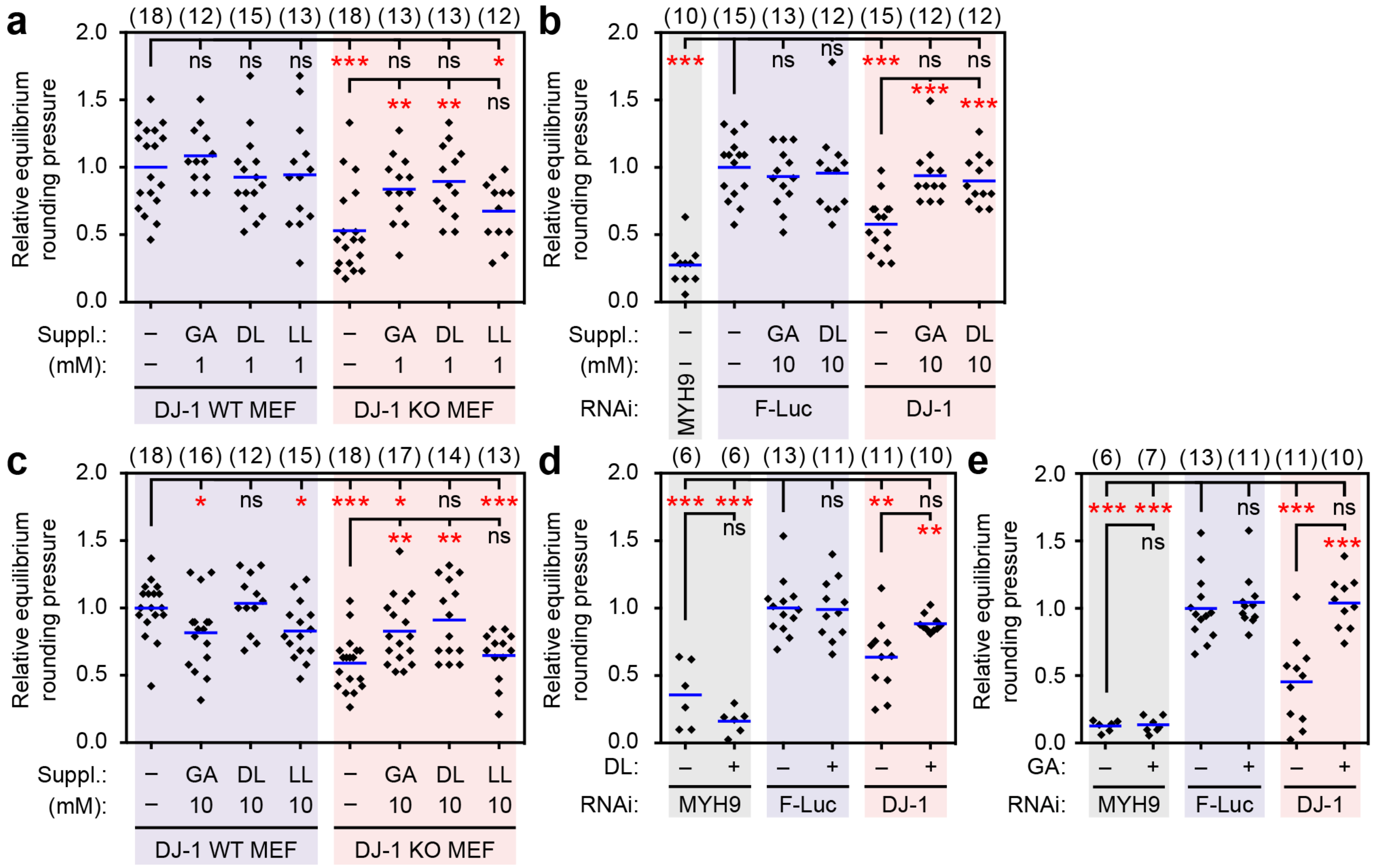

Supplementary Figure 10. Glycolate and D-lactate rescue the rounding pressure defect of mitotic mammalian cells defective in Parkinson's-related gene DJ-1. (**a**) Equilibrium rounding pressure of mitotic WT and DJ-1 knockout (KO) MEF cells incubated with 1 mM glycolate (GA), D-lactate (DL), or L-lactate (LL) for one hour. (**b**) Equilibrium rounding pressure of mitotic HeLa cells incubated with 10 mM GA or DL for one hour. (**c**) Equilibrium rounding pressure of mitotic WT and DJ-1 KO MEF cells incubated with 10 mM GA, DL, or LL for one hour. (**d**) Equilibrium rounding pressure of mitotic RNAi-treated HeLa cells. Measurements were carried out before (-) and after (+) one hour of addition of 1 mM DL. (**e**) Equilibrium rounding pressure of mitotic RNAi-treated HeLa cells. Measurements were carried out before (-) and after (+) one hour of addition of 1 mM GA. Values relative to firefly luciferase (F-Luc) control data average are shown. Each diamond corresponds to one cell. '(*n*)' denotes number of cells measured. Blue bars, mean. Statistical significance was determined by Mann-Whitney U test (ns, $p > 0.05$; *, $p \le 0.05$; **, $p < 0.01$, ***, $p < 0.001$).

Supplementary Table 1

Symbol	Short name	Primary screen	Secondary screen			Category
		Force	Force	Pressure	Volume	
ACTN4	alpha-actinin 4	0.63	0.69	0.67	1.00	CM[10]
ATR	ATR serine/threonine kinase	0.74	0.75	0.73	0.98	CC[11]
CAMK2A	calcium/calmodulin-dependent protein kinase	0.69	0.64	0.71	0.96	NO
CDC37	cell division cycle 37	0.46	0.56	0.51	0.95	NO (CC[12])
CDK11A	cyclin-dependent kinase 11A	0.65	0.52	0.59	0.93	CC[13,14]*
CEP72	centrosomal protein 72kDa	0.83	0.41	0.40	1.01	CC[15]
CERK	ceramide kinase	0.78	0.64	0.70	0.95	NO
CLEC16A	c-type lectin domain family 16, member A	0.73	0.67	0.62	0.96	NO (CC*)
CYFIP1	cytoplasmic FMR1 interacting protein 1	0.67	0.83	0.64	0.98	CM[16]
DFNB31	deafness, autosomal recessive 31	0.69	0.52	0.57	0.90	NO (CM[17])
DIAPH1	diaphanous-related formin 1	0.66	0.33	0.35	0.98	MCR[5,18]
F2R	coagulation factor II (thrombin) receptor	0.67	0.44	0.40	1.04	CM[19]
FAM134A	family with sequence similarity 134, member	0.65	0.57	0.60	0.99	NO (CC*)
FGF2	fibroblast growth factor 2 (basic)	0.56	0.68	0.52	0.98	CM[20]
GNA13	guanine nucleotide binding protein, alpha 13	0.48	0.44	0.59	0.88	NO (CM[21])
KCNJ15	potassium inwardly-rectifying channel	0.77	0.72	0.67	1.04	NO
MYH7B	myosin, heavy chain 7B, cardiac muscle, beta	0.38	0.63	0.69	0.97	CM[22]
**MYH9**	myosin, heavy chain 9, non-muscle	0.35	0.20	0.15	1.14	MCR*[4,18]
MYL12B	myosin, light chain 12B, regulatory	0.27	0.31	0.28	1.00	CM[23]
MYRIP	myosin VIIA and rab interacting protein	0.74	0.64	0.64	1.00	NO
DJ-1	parkinson protein 7	0.55	0.48	0.57	0.94	NO (CC[24,25])
PHKG2	phosphorylase kinase, gamma 2 (testis)	0.60	0.67	0.61	1.04	NO
PIK3CB	phosphatidylinositol-4,5-bisphosphate 3-kinase	0.80	0.66	0.68	0.98	CM[26]
PIM2	pim-2 oncogene, serine/threonine kinase	0.78	0.61	0.63	0.97	CC[27]
RAC1	ras-related C3 botulinum toxin substrate 1	0.71	0.38	0.39	0.97	CM[28]
ROCK2	rho-associated kinase 2	0.61	0.68	0.70	0.98	MCR[18,29]
SEPT1	septin 1	0.60	0.61	0.57	1.01	CM[30,31] (CC*)
SEPT9	septin 9	0.62	0.61	0.62	0.96	CM[30,31]
SKA1	spindle and kinetochore associated complex	0.71	0.52	0.63	0.93	CC*
SOS2	son of sevenless homolog 2 (Drosophila)	0.65	0.32	0.35	0.94	NO
TAF5	TAF5 RNA polymerase II, associated factor	0.50	0.42	0.53	0.95	NO
VAV2	vav 2 guanine nucleotide exchange factor	0.60	0.66	0.56	0.98	CM[32,33]
VAV3	vav 3 guanine nucleotide exchange factor	0.57	0.39	0.47	0.92	CM[33]
WAS	wiskott-aldrich syndrome	0.28	0.62	0.59	1.05	CM[34]

BCAR1	breast cancer anti-estrogen resistance 1	0.62	0.73	0.83	0.94	CM[35]
BUB1	BUB1 mitotic checkpoint serine/threonine	0.69	0.81	0.81	0.97	CC[36]*
CHRM3	cholinergic receptor, muscarinic 3	0.70	0.50	0.59	0.94	NO
CLIC5	chloride intracellular channel 5	0.79	0.67	0.82	0.95	NO (CM[37])
DBN1	drebrin 1	0.59	0.67	0.74	0.95	CM[38]
DLG4	discs, large homolog 4 (Drosophila)	0.74	0.72	0.76	0.97	NO
DLG5	discs, large homolog 5 (Drosophila)	0.61	0.67	0.87	0.89	NO (CM[39])
ECT2	epithelial cell transforming 2	0.46	0.67	0.66	0.98	MCR*[18,40]
FSCN1	fascin actin-bundling protein 1	0.28	0.72	0.76	0.99	CM[41]
MKNK2	MAP kinase interacting serine/threonine kinase	0.66	0.76	0.86	0.95	NO
**MYH1**	myosin, heavy chain 1, skeletal muscle, adult	0.61	0.75	0.87	0.90	CM[42]
**PFN1**	profilin 1	0.65	0.54	0.69	0.93	CM[43]
RBKS	ribokinase	0.75	0.75	0.86	0.95	NO
TMEM63B	transmembrane protein 63B	0.70	0.51	0.60	1.02	NO (CC*)
UACA	uveal autoantigen	0.67	0.62	0.73	0.96	CM[44](CC*)

Supplementary Table 1. Alphabetical list of all mitotic cell mechanics genes. Top section, mitotic cell mechanics genes leading to decreased force and pressure according to the criteria specified in the Methods. Bottom section, mitotic cell mechanics genes leading to decreased force. Bold, genes leading to change in volume. Shown are common gene symbols (Symbol), short version of gene name (short name), primary screen force results and secondary screen force, pressure and volume results as well as a classification into the following categories (category, with selected references): MCR – Reported role in regulation and/or mechanics of mitotic cell rounding; CM – Reported role in cell mechanics without association in mitotic cell mechanics (cell motility and migration, cytoskeletal remodeling, cell adhesion, muscle contraction); CC – Reported role in cell cycle regulation, cell survival and proliferation without impact on cell mechanics; NO – No reported role in cell cycle regulation or cell mechanics found, partially with unknown function. Categories in brackets represent possible association based on similarity to related genes and postulated roles without solid reported evidence. * indicates genes reported to be required for correct cell division in earlier genome-wide esiRNA screens [7,8]. Force, pressure and volume data are averages relative to control cells.

Supplementary References

1 Keen, N. & Taylor, S. Aurora-kinase inhibitors as anticancer agents. *Nat Rev Cancer* **4**, 927-936, (2004).

2 Lenart, P. *et al*. The small-molecule inhibitor BI 2536 reveals novel insights into mitotic roles of polo-like kinase 1. *Curr Biol* **17**, 304-315, (2007).

3 Steegmaier, M. *et al*. BI 2536, a potent and selective inhibitor of polo-like kinase 1, inhibits tumor growth *in vivo*. *Curr Biol* **17**, 316-322, (2007).

4 Stewart, M. P. *et al*. Wedged AFM-cantilevers for parallel plate cell mechanics. *Methods* **60**, 186-194, (2013).

5 Cattin, C. J. *et al*. Mechanical control of mitotic progression in single animal cells. *Proc Natl Acad Sci U S A* **112**, 11258-11263, (2015).

6 Fischer-Friedrich, E., Hyman, A. A., Julicher, F., Muller, D. J. & Helenius, J. Quantification of surface tension and internal pressure generated by single mitotic cells. *Sci Rep* **4**, 6213, (2014).

7 Kittler, R. *et al*. Genome-scale RNAi profiling of cell division in human tissue culture cells. *Nat Cell Biol* **9**, 1401-1412, (2007).

8 Neumann, B. *et al*. Phenotypic profiling of the human genome by time-lapse microscopy reveals cell division genes. *Nature* **464**, 721-727, (2010).

9 Poser, I. *et al*. BAC TransgeneOmics: a high-throughput method for exploration of protein function in mammals. *Nat Methods* **5**, 409-415, (2008).

10 Honda, K. *et al*. Actinin-4, a novel actin-bundling protein associated with cell motility and cancer invasion. *J Cell Biol* **140**, 1383-1393, (1998).

11 Cliby, W. A. *et al*. Overexpression of a kinase-inactive ATR protein causes sensitivity to DNA-damaging agents and defects in cell cycle checkpoints. *EMBO J* **17**, 159-169, (1998).

12 Stepanova, L., Leng, X., Parker, S. B. & Harper, J. W. Mammalian p50Cdc37 is a protein kinase-targeting subunit of Hsp90 that binds and stabilizes Cdk4. *Genes Dev* **10**, 1491-1502, (1996).

13 Hu, D., Mayeda, A., Trembley, J. H., Lahti, J. M. & Kidd, V. J. CDK11 complexes promote pre-mRNA splicing. *J Biol Chem* **278**, 8623-8629, (2003).

14 Malumbres, M. *et al*. Cyclin-dependent kinases: a family portrait. *Nat Cell Biol* **11**, 1275-1276, (2009).

15 Oshimori, N., Li, X., Ohsugi, M. & Yamamoto, T. Cep72 regulates the localization of key centrosomal proteins and proper bipolar spindle formation. *EMBO J* **28**, 2066-2076, (2009).

16 Chen, Z. *et al*. Structure and control of the actin regulatory WAVE complex. *Nature* **468**, 533-538, (2010).

17 Mburu, P. *et al*. Defects in whirlin, a PDZ domain molecule involved in stereocilia elongation, cause deafness in the whirler mouse and families with DFNB31. *Nat Genet* **34**, 421-428, (2003).

18 Ramanathan, S. P. *et al*. Cdk1-dependent mitotic enrichment of cortical myosin II promotes cell rounding against confinement. *Nat Cell Biol* **17**, 148-159, (2015).

19 Kahn, M. L., Nakanishi-Matsui, M., Shapiro, M. J., Ishihara, H. & Coughlin, S. R. Protease-activated receptors 1 and 4 mediate activation of human platelets by thrombin. *J Clin Invest* **103**, 879-887, (1999).

20 Kottakis, F. *et al*. FGF-2 regulates cell proliferation, migration, and angiogenesis through an NDY1/KDM2B-miR-101-EZH2 pathway. *Mol Cell* **43**, 285-298, (2011).

21 Simoncini, T. *et al*. Estrogen receptor alpha interacts with Galpha13 to drive actin remodeling and endothelial cell migration via the RhoA/Rho kinase/moesin pathway. *Mol Endocrinol* **20**, 1756-1771, (2006).

22 Desjardins, P. R., Burkman, J. M., Shrager, J. B., Allmond, L. A. & Stedman, H. H. Evolutionary implications of three novel members of the human sarcomeric myosin heavy chain gene family. *Mol Biol Evol* **19**, 375-393, (2002).

23 Iwasaki, T., Murata-Hori, M., Ishitobi, S. & Hosoya, H. Diphosphorylated MRLC is required for organization of stress fibers in interphase cells and the contractile ring in dividing cells. *Cell Struct*

Funct **26**, 677-683, (2001).
24 Taira, T. *et al*. DJ-1 has a role in antioxidative stress to prevent cell death. *EMBO Rep* **5**, 213-218, (2004).
25 Toyoda, Y. *et al*. Products of the Parkinson's disease-related glyoxalase DJ-1, D-lactate and glycolate, support mitochondrial membrane potential and neuronal survival. *Biol Open* **3**, 777-784, (2014).
26 Heller, R. *et al*. Overlapping and distinct roles for PI3Kbeta and gamma isoforms in S1P-induced migration of human and mouse endothelial cells. *Cardiovasc Res* **80**, 96-105, (2008).
27 Morishita, D., Katayama, R., Sekimizu, K., Tsuruo, T. & Fujita, N. Pim kinases promote cell cycle progression by phosphorylating and down-regulating p27Kip1 at the transcriptional and posttranscriptional levels. *Cancer Res* **68**, 5076-5085, (2008).
28 Das, T. *et al*. A molecular mechanotransduction pathway regulates collective migration of epithelial cells. *Nat Cell Biol* **17**, 276-287, (2015).
29 Maddox, A. S. & Burridge, K. RhoA is required for cortical retraction and rigidity during mitotic cell rounding. *J Cell Biol* **160**, 255-265, (2003).
30 Bridges, A. A. & Gladfelter, A. S. Septin Form and Function at the Cell Cortex. *J Biol Chem* **290**, 17173-17180, (2015).
31 Kinoshita, M. The septins. *Genome Biol* **4**, 236, (2003).
32 Abe, K. *et al*. Vav2 is an activator of Cdc42, Rac1, and RhoA. *J Biol Chem* **275**, 10141-10149, (2000).
33 Bhavsar, P. J., Vigorito, E., Turner, M. & Ridley, A. J. Vav GEFs regulate macrophage morphology and adhesion-induced Rac and Rho activation. *Exp Cell Res* **315**, 3345-3358, (2009).
34 Cory, G. O., Garg, R., Cramer, R. & Ridley, A. J. Phosphorylation of tyrosine 291 enhances the ability of WASp to stimulate actin polymerization and filopodium formation. Wiskott-Aldrich Syndrome protein. *J Biol Chem* **277**, 45115-45121, (2002).
35 Abassi, Y. A., Rehn, M., Ekman, N., Alitalo, K. & Vuori, K. p130Cas Couples the tyrosine kinase Bmx/Etk with regulation of the actin cytoskeleton and cell migration. *J Biol Chem* **278**, 35636-35643, (2003).
36 Elowe, S. Bub1 and BubR1: at the interface between chromosome attachment and the spindle checkpoint. *Mol Cell Biol* **31**, 3085-3093, (2011).
37 Singh, H., Cousin, M. A. & Ashley, R. H. Functional reconstitution of mammalian 'chloride intracellular channels' CLIC1, CLIC4 and CLIC5 reveals differential regulation by cytoskeletal actin. *FEBS J* **274**, 6306-6316, (2007).
38 Geraldo, S., Khanzada, U. K., Parsons, M., Chilton, J. K. & Gordon-Weeks, P. R. Targeting of the F-actin-binding protein drebrin by the microtubule plus-tip protein EB3 is required for neuritogenesis. *Nat Cell Biol* **10**, 1181-1189, (2008).
39 Wakabayashi, M. *et al*. Interaction of lp-dlg/KIAA0583, a membrane-associated guanylate kinase family protein, with vinexin and beta-catenin at sites of cell-cell contact. *J Biol Chem* **278**, 21709-21714, (2003).
40 Matthews, H. K. *et al*. Changes in Ect2 localization couple actomyosin-dependent cell shape changes to mitotic progression. *Dev Cell* **23**, 371-383, (2012).
41 Yamashiro-Matsumura, S. & Matsumura, F. Purification and characterization of an F-actin-bundling 55-kilodalton protein from HeLa cells. *J Biol Chem* **260**, 5087-5097, (1985).
42 Leinwand, L. A., Saez, L., McNally, E. & Nadal-Ginard, B. Isolation and characterization of human myosin heavy chain genes. *Proc Natl Acad Sci U S A* **80**, 3716-3720, (1983).
43 Ding, Z., Gau, D., Deasy, B., Wells, A. & Roy, P. Both actin and polyproline interactions of profilin-1 are required for migration, invasion and capillary morphogenesis of vascular endothelial cells. *Exp Cell Res* **315**, 2963-2973, (2009).
44 Mori, Y., Matsui, T., Omote, D. & Fukuda, M. Small GTPase Rab39A interacts with UACA and regulates the retinoic acid-induced neurite morphology of Neuro2A cells. *Biochem Biophys Res Commun* **435**, 113-119, (2013).
