# Supplementary material for: Genome-scale single-cell mechanical phenotyping reveals disease-related genes involved in mitotic rounding
Source: Nat Commun. 2017 Nov 2;8:1266. doi: 10.1038/s41467-017-01147-6 (PMC5668354; doi:10.1038/s41467-017-01147-6)
Supplement: Supplementary file 2 — Description of Additional Supplementary Files [file 41467_2017_1147_MOESM2_ESM.pdf]

Description of Additional Supplementary Files

File Name: Supplementary Data 1
Description: Supplementary Data 1 shows the list of the genes targeted in the mitotic cell mechanics screen. Information on target gene (gene name(s), description and ENSEMBLE ID), esiRNA (ID), the mitotic rounding force relative to that of control RNAi cells transfected with esiRNA against firefly luciferase (F-Luc), and evaluation of each round of results are shown. Background coloring: blue and orange, lower and higher rounding force, respectively; green, significant statistical difference to F-Luc RNAi control. For columns entitled "Evaluation": LO (HI), lower (higher) rounding force, respectively; "-", no significantly altered rounding force. Criteria for the evaluation are detailed in the Methods.

File Name: Supplementary Data 2
Description: Supplementary Data 2 shows the list of the primary hit genes the silencing of which significantly altered rounding force.

File Name: Supplementary Data 3
Description: Supplementary Data 3 shows the list of the genes tested in the secondary screen. Relative rounding force, pressure and volume are shown. Background coloring: blue and orange, lower and higher rounding force, pressure, or volume, respectively; green, significant statistical difference to F-Luc RNAi control. For columns entitled "Evaluation": LO (HI) – F/P/V, lower (higher) rounding force, pressure, or volume, respectively; "-", no significantly altered rounding force, pressure, or volume.

File Name: Supplementary Data 4
Description: Supplementary Data 4 shows the list of the 49 mitotic cell mechanics genes constituting the main result of the present screen.

File Name: Supplementary Movie 1
Description: The representative movie shows a control RNAi cell progressing through mitosis. The cell stably expressing MYH9-EGFP (green) and histone H2B-mCherry (red) was confined at a height of 10 µm by a wedged microcantilever held parallel to the substrate. For details of this parallel plate configuration see Supplementary Fig. 4c. The movie plays 600-times faster (one second in the movie equals to ten minutes in real time). Scale bar, 20 µm.

File Name: Supplementary Movie 2
Description: The representative movie shows a FAM134A RNAi cell progressing through mitosis. The cell stably expressing MYH9-EGFP (green) and histone H2B-mCherry (red) was confined at a height of 10 µm by a wedged microcantilever held parallel to the substrate. For details of this parallel plate configuration see Supplementary Fig. 4c. The movie plays 600-times faster (one second in the movie equals to ten minutes in real time). Note the defective cortical targeting of MYH9-EGFP and the prometaphase delay. Scale bar, 20 µm.
